# A novel hermit crab optimization algorithm

**DOI:** 10.1038/s41598-023-37129-6

**Published:** 2023-06-19

**Authors:** Jia Guo, Guoyuan Zhou, Ke Yan, Binghua Shi, Yi Di, Yuji Sato

**Affiliations:** 1 School of Information Engineering, Hubei University of Economics, Wuhan, 430205 China; 2 Hubei Internet Finance Information Engineering Technology Research Center, Wuhan, 430205 China; 3 China Construction Third Engineering Bureau Installation Engineering Co., Ltd., Wuhan, 430075 China; 4 Faculty of Computer and Information Sciences, Hosei University, Tokyo, 184-8584 Japan

**Keywords:** Evolution, Engineering

## Abstract

High-dimensional optimization has numerous potential applications in both academia and industry. It is a major challenge for optimization algorithms to generate very accurate solutions in high-dimensional search spaces. However, traditional search tools are prone to dimensional catastrophes and local optima, thus failing to provide high-precision results. To solve these problems, a novel hermit crab optimization algorithm (the HCOA) is introduced in this paper. Inspired by the group behaviour of hermit crabs, the HCOA combines the optimal search and historical path search to balance the depth and breadth searches. In the experimental section of the paper, the HCOA competes with 5 well-known metaheuristic algorithms in the CEC2017 benchmark functions, which contain 29 functions, with 23 of these ranking first. The state of work BPSO-CM is also chosen to compare with the HCOA, and the competition shows that the HCOA has a better performance in the 100-dimensional test of the CEC2017 benchmark functions. All the experimental results demonstrate that the HCOA presents highly accurate and robust results for high-dimensional optimization problems.

## Introduction

There are many basic laws of existence in nature, such as cooperation and competition, genetic variation, and survival of the fittest. In recent years, some scholars have been inspired by nature-based processes and applied these inspirations to the domain of computing. Genetic algorithms (GAs)^1^ are inspired by the process of biological evolution through selection, inheritance and mutation. Differential evolution (DE)^2^ is inspired by the process of cooperation and competition among individuals in a biological population. DE converges faster and more accurately in minimizing possible nonlinear and nondifferentiable continuous space function problems. The group search optimizer (GSO)^3^ is inspired by the process of production and consumption. GSO performs better than the other algorithms in a multimodal benchmark function with a few local minima.

Similarly, some researchers have proposed optimization algorithms inspired by animal behaviour to solve optimization problems. Kennedy^4^ proposed particle swarm optimization (PSO), which is inspired by the social behaviour of foraging birds, to effectively pursue the optimization of nonlinear functions in multidimensional space. To better optimize multivariable functions, Karaboga^5^ proposed an artificial bee colony algorithm (ABC) inspired by the honey bees social behaviour. ABC is used only to optimize 10-, 20- and 30-dimensional functions. The firefly algorithm (FA)^6^ utilizes the influence of light on fireflies, and the FA shows a significantly improved performance over PSO in multimodal optimization problems. The cuckoo search optimization^7^ mimics the parasitic behaviour of the cuckoo bird. The chicken swarm optimization (CSO)^8^ simulates the hierarchical structure of chicken flocks and the foraging behaviour of chickens, including roosters and hens. The dragonfly algorithm (DA)^9^ simulates the survival behaviour of dragonflies, including separation, parade, aggregation, predation and escape. DA lacks a width search for high-dimensional space, so it performs poorly in high-dimensional optimization problems. The DA algorithm is superior to other algorithms for optimizations in 30 dimensions. The lion optimization algorithm (LOA)^10^ is an algorithm inspired by a simulation of lions’ behaviours of solitude and cooperation. The LOA outperforms other optimization algorithms in only 30 dimensions of the benchmark function, and it tends to fall into a local optimum prematurely in high-dimensional problems. Inspired by the process of finding the shortest distance between ants’ food and their residence, Dorigo^11^ proposed the ant colony optimization algorithm (ACO). The whale optimization algorithm (WAO)^12^ simulates the hunting of prey, prey envelopment, and the bubble net hunting behaviour of humpback whales. There are also some nature-inspired and animal-inspired algorithms that are extensively used by researchers in various fields, such as path design^13–15^, control autoregressive models^16–19^ and urban development^20,21^.

According to the no free lunch theorem, every optimization problem cannot be solved by only one algorithm. Therefore, it is necessary to develop or improve additional metaheuristic optimization algorithms to address different types of optimization problems. A high-dimensional optimization problem is a typical representative of an optimization problem. With the continuous development of blockchain technology^22–25^, big data^26,27^ and practical nanotechnology^28–30^, the dimensionality of optimization problems is increasing dramatically. Li^31^ proposed a dimension dynamic sine cosine algorithm (DDSCA). In the DDSCA, the solution of each dimension is obtained first, and then the greedy algorithm is used to combine the solution of other dimensions and form a new solution. Yang^32^ introduced an elitist oriented particle swarm optimization algorithm (EDPSO), which uses historical information about particles to efficiently solve high-dimensional optimization problems. Chen^33^ designed an Efficient hierarchical surrogate assisted differential evolution (EHSDE), which balances exploration and development in a high-dimensional optimized space using a hierarchical approach. On the one hand, the above algorithms cannot effectively balance between depth searches and breadth searches in high-dimensional spaces. On the other hand, these algorithms cannot jump to the local optimum in the initial stages of the search, or are unable to search for a preferable value after jumping out of the local optimum. So, it is also crucial to develop a new optimization algorithm to solve high-dimensional optimization problems as effectively as possible.

This paper introduces a new optimization algorithm, which is named hermit crab optimization algorithm (HCOA), to solve high-dimensional optimization problems. It is inspired by the distinctive behaviour of hermit crabs in searching for, and changing to, appropriate houses to survive during their continuous growth. More specifically, the main research contributions of this paper are as follows:

1. Optimal search: The hermit crabs search in the vicinity of the alpha hermit crab of the entire population. In adherence to this rule, HCAO guarantees the accuracy of the search.
2. Historical path search: The hermit crabs search around the historical path of the population’s alpha hermit crabs. With this strategy, the HCOA balances between breadth and depth searches in a high-dimensional space, and helps the HCOA to jump out of the local optimum.

The remaining sections of the manuscript are organized as follows. “Materials and methods” elaborates on the proposed algorithm in detail. “Results” shows the details and results of the simulation experiments. “Conclusions” concludes this work and presents future works.

## Materials and methods

### Behaviour of hermit crab

Hermit crabs are arthropods similar to shrimp and crabs that live mainly in coastal areas. They are also omnivorous and are known as the “scavengers” of the seashore, eating everything from algae and food scraps to parasites, and they play an essential role in the ecological balance. However, hermit crabs rely heavily on their houses for survival, and years of research have shown that proper houses help hermit crabs survive, feed and resist predators, and if hermit crabs lose their houses, the soft tissue structures of their abdomens become exposed and unprotected. Hermit crabs may die if they live in unsuitable houses or have no houses to live in for a long time. As they grow, hermit crabs are continuously searching and acquiring houses that are appropriate for their survival. Its population behaviour of searching for, and changing to, new houses is a unique natural process. The hermit crabs search for a proper house to survive in their surrounding location or host an aged house that other crabs have shed. If the hermit crab is unable to find a suitable new house, it must return to its original house.

### Hermit crab optimization algorithm

**Figure 1:**
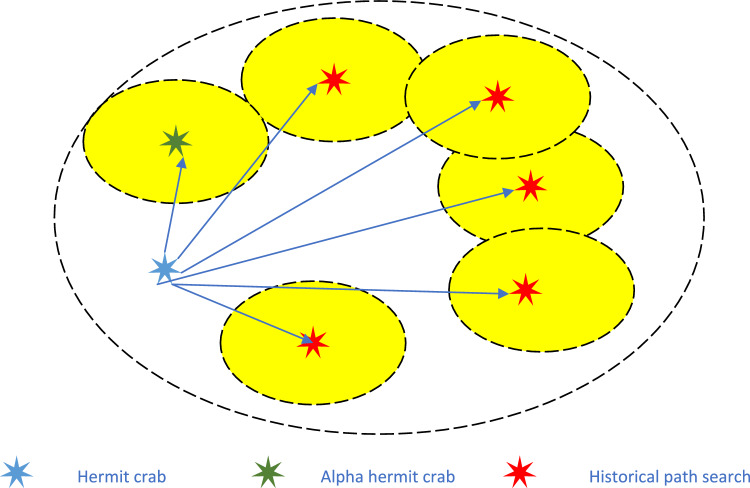
The search diagram for the HCOA.

**Figure 2:**
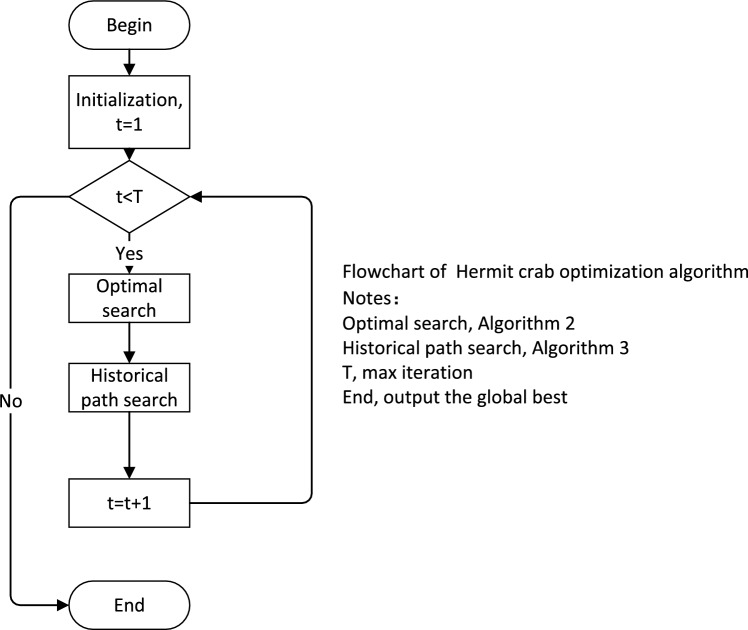
The flowchart of the HCOA.

Inspired by the constant house-searching and house-changing behaviour of hermit crabs, we idealize the characteristics of hermit crabs’ behaviours. Relating the process of hermit crabs’ house searching and house changing to the objective function to be optimized, we are able to design a hermit crab-inspired optimization algorithm (the HCOA). In hermit crab populations, there are many factors involved in selecting the right house, including size, species and colour. In the HCOA, for simplicity, we assume that each hermit crab has no mass or volume, and represents only a point in space, and each point is a solution to a certain problem. The fitness of each hermit crab for a new house is associated with the optimal value of the target function, and an adaptation degree analogy is associated with the house. Because of the large and variable distribution of crustaceans in coastal areas, we randomly generate a large number of houses in the HCOA. Based on the behaviour of the hermit crabs, we use two house-searching and house-changing rules, which are denoted as the optimal search and the historical path search. These two strategies can help the HCOA balance the breadth searches and width searches in a high-dimensional search space, and increase the possibility of jumping out of a local optimum. The search diagram for the HCOA is shown in Fig. 1. At the same time, the basic steps of the HCOA are summarized using the pseudocode displayed in algorithm 1, and the HCOA flowchart is displayed in Fig. 2. The two the HCOA search strategies create only linear transformations in the time complexity. Therefore, the time complexity of an the HCOA is still linear complexity *O*(*n*).

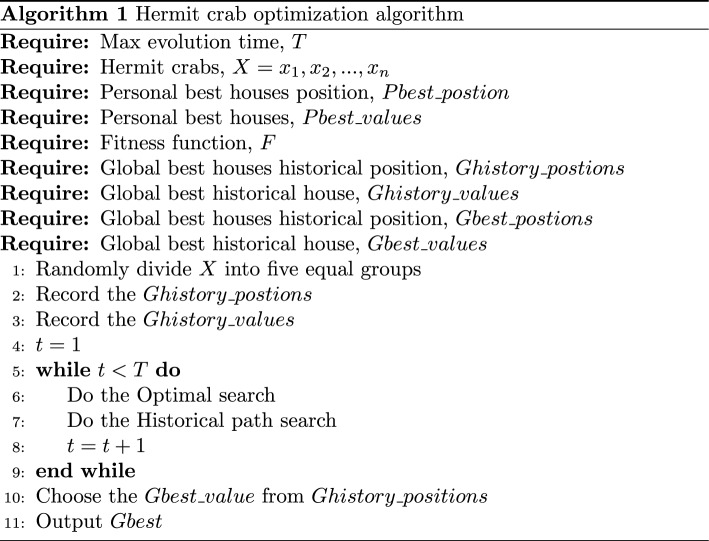



### Optimal search

The alpha hermit crab of the crab population gains more valuable survival experience than the other hermit crabs, and it is more experienced in finding a new house. Therefore, other hermit crabs are more likely to find more appropriate houses in the vicinity of the population’s alpha hermit crab. If other hermit crabs find a more appropriate house than the one it currently has, it changes houses. By comparison, if it does not find a more suitable house, it continues to use the original house in order to survive. In the HCOA, after each calculation of the function fitness, the fitness of all the hermit crabs is ranked. The hermit crab with the best fitness is selected for comparison with the alpha hermit crab. If a hermit crab with the best fitness is better, then it is more experienced in survival than the existing alpha hermit crab. The optimal search process is summarized in the pseudocode shown in Algorithm 2. With the guidance of this rule, the HCOA can accurately find the optimal solution.

1 $$\begin{aligned} \alpha &= (Alpha^{t}+Pbest^{t})/2 \nonumber \\ \delta &= \Vert Alpha^{t}-Pbest^{t}\Vert \nonumber \\ Pcandidate^{t+1}(\gamma )&= GD(\alpha ,\delta ) \end{aligned}$$

In the (*t*)th generation, the alpha hermit crab finds the most appropriate house position $Alpha^t$. $Pbest^{t}(\gamma )$ means that each hermit crab in the population finds the (*t*)th most appropriate house’s position, and $Pcandidate^{t+1}(\gamma )$ is the $(t + 1)$th candidate house’s position. $GD(\alpha ,\delta )$ is a Gaussian distribution with mean $\alpha$ and standard deviation $\delta$, which is used to simulate the distribution of the houses.
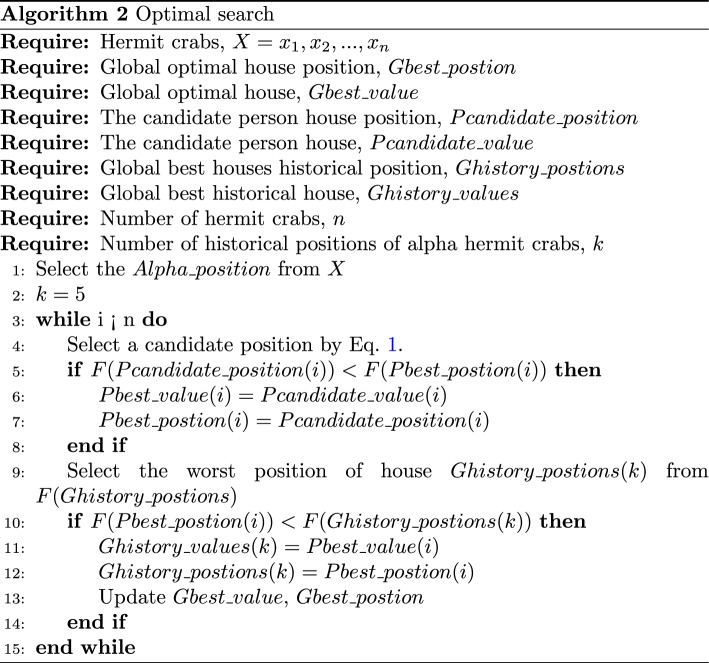


### Historical path search

The alpha hermit crab from the entire crab population is replaced when other hermit crabs have a more appropriate house than the alpha hermit crab. However, each generation of alpha hermit crabs in the population sheds its original house when it seeks a more appropriate house. The original house remains in place, while the alpha hermit crab replaces it with a more appropriate house. These original houses may be used by other hermit crabs. It is also possible that more appropriate houses exist around these houses for other hermit crabs to live in. The houses abandoned by alpha hermit crabs change with the environment and hermit crab behaviours. On the one hand, they may simply disappear; on the other hand, they may appear near their original location. Therefore, other hermit crabs want to find a more suitable house. In the HCOA, other hermit crabs search around the historical path of the five houses where the alpha hermit crab has recently left, because there may be a better chance of finding a house that suits them. A historical path means that the HCOA has deeper search spaces. A hermit crab may find a better house nearby on the five historical paths and attain a house replacement. This search process increases the HCOA width search in high-dimensional space. If a more suitable shell is not found, the hermit crab returns to its original shell. The historical path search process can be summarized in the pseudocode shown in Algorithm 3.

2 $$\begin{aligned} \beta&= (Pbest^{t}(\gamma )+Ghistory^{t}(\omega ))/2 \nonumber \\ \lambda&= \Vert Pbest^{t}((\gamma ))-Ghistory^{t}(\omega )\Vert \nonumber \\ Candiate\_pbest^{t+1}(\gamma ,\omega )&= GD(\beta ,\lambda ) \end{aligned}$$

3 $$\begin{aligned} Pbest^{t+1}(\gamma )&= \left\{ \begin{aligned}&Candidate\_pbest^{t+1}(\gamma , \omega ),if(\text {F(}Pbest^{t})>F(Candidate\_pbest^{t+1}(\gamma , \omega )) \\&Pbest^{t}(\gamma ),else \\ \end{aligned} \right. \end{aligned}$$

In the (*t*)th generation, the best personal position for each hermit crab is $Pbest^{t}(\gamma )$. By the definition of the HCOA, $\omega =(1, 5)$ is the alpha hermit crab in most recent history to shed the first few houses, and the population’s current alpha hermit crabs keep the houses it recently replaced. We use $Ghistory^{t}(\omega )$ to record the population’s alpha hermit crabs houses’ historical position. $Candiate\_pbest^{t+1}(\gamma , \omega )$ means hermit crabs search around the $\omega$ houses’ positions. $GD(\beta ,\lambda )$ is a Gaussian distribution with mean $\beta$ and standard $\lambda$, which is used to simulate the distribution of houses. *F* is the indicator test function.
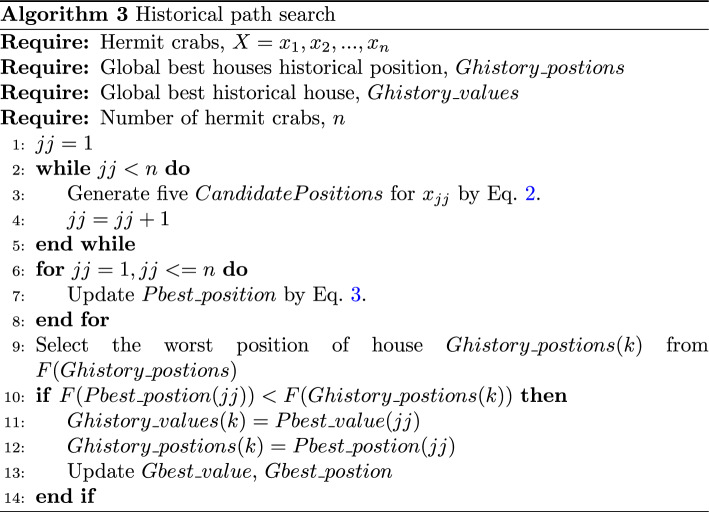


## Results

### Experimental methods

To reflect the comprehensive performance of the HCOA, we choose the CEC2017 benchmark function^34^. The CEC2017 benchmark function includes a unimodal function ($f1-f2$), simple multipeak function ($f3-f9$), hybrid function ($f10-f19$) and composition function ($f20-f29$). The test dimensions are 10, 30, 50 and 100. The highest dimension of 100 recommended in the CEC2017 benchmark function is chosen to show the reasonableness of the experiment. Five well-known parameter-free mate-heuristics, BBPSO^35^, PBBPSO^36^, DLSBBPSO^37^, TBBPSO^38^ and ETBBPSO^39^ are used as comparison groups. To reduce the impact of chance errors on the experimental results, all the trials are attempted 37 times. All the algorithms have a population size of 100 and a maximum number of iterations of 1.00E+4 and use the same settings as in the original paper.

### Experimental results

To better analyse the experimental results, GT is used to measure the performance of each algorithm. In this work, GT is defined as $\left\| Gobaloptimum-theoreticaloptimum \right\|$.

Specific numerical results, including the mean value (MV) and standard deviation (Std) of 37 independent runs, are displayed in Tables 1 and 2. The Friedman statistic test is used to analyse the results. The rank results (RRs) are also shown in Tables 1 and 2. The average rank point of the HCOA is 1.4828, which is 56.121% better than the second ranked algorithm BBPSO. the HCOA provides a solution to high-dimensional optimization problems. The average ranks are shown at the bottom of Table 2. The results of the first ranking of the HCOA out of 29 benchmarking functions in CEC2017 are in Table 3, and the remaining ranking results are in Table 4.

**Table 1:** Experimental results, of the HCOA, DLSBBPSO, TBBPSO, BBPSO, ETBBPSO and PBBPSO for $f_1 - f_{15}$.

Number	Data Type	the HCOA	DLSBBPSO	TBBPSO	BBPSO	ETBBPSO	PBBPSO
$f_{1}$	MV	3.645E+04	1.580E+04	2.426E+04	2.273E+04	2.603E+04	5.388E+04
	Std	5.445E+04	2.387E+04	4.355E+04	2.667E+04	2.494E+04	5.855E+04
	RR	5	1	3	2	4	6
$f_{2}$	MV	2.707E+90	1.775E+134	1.749E+128	1.433E+120	1.216E+127	4.831E+163
	Std	1.647E+91	1.079E+135	1.064E+129	6.069E+120	7.393E+127	6.554E+04
	RR	1	5	4	2	3	6
$f_{3}$	MV	8.421E+05	3.389E+06	1.905E+06	3.728E+06	3.452E+06	3.399E+06
	Std	4.333E+05	3.313E+06	8.890E+05	3.067E+06	2.698E+06	2.192E+06
	RR	1	3	2	6	5	4
$f_{4}$	MV	1.331E+02	1.549E+02	1.649E+02	1.492E+02	1.597E+02	1.778E+02
	Std	5.133E+01	4.537E+01	5.057E+01	5.027E+01	4.861E+01	5.785E+01
	RR	1	3	5	2	4	6
$f_{5}$	MV	7.102E+02	9.269E+02	9.322E+02	8.480E+02	8.858E+02	9.306E+02
	Std	1.438E+02	1.755E+02	1.866E+02	1.906E+02	1.618E+02	1.673E+02
	RR	1	4	6	2	3	5
$f_{6}$	MV	2.901E+01	4.426E+01	3.821E+01	3.746E+01	3.870E+01	4.129E+01
	Std	7.219E+00	1.043E+01	7.312E+00	8.049E+00	7.898E+00	6.479E+00
	RR	1	6	3	2	4	5
$f_{7}$	MV	8.560E+02	8.442E+02	8.785E+02	9.159E+02	8.960E+02	9.333E+02
	Std	1.552E+02	1.394E+02	1.378E+02	1.910E+02	1.372E+02	1.278E+02
	RR	2	1	3	5	4	6
$f_{8}$	MV	7.863E+02	8.330E+02	9.245E+02	8.944E+02	8.495E+02	8.996E+02
	Std	1.464E+02	1.719E+02	1.686E+02	1.843E+02	1.783E+02	1.670E+02
	RR	1	2	6	4	3	5
$f_{9}$	MV	3.024E+04	2.886E+04	3.903E+04	3.539E+04	2.970E+04	4.121E+04
	Std	7.992E+03	1.924E+04	1.240E+04	8.117E+03	7.882E+03	3.221E+04
	RR	3	1	5	4	2	6
$f_{10}$	MV	2.208E+04	2.946E+04	2.416E+04	2.457E+04	2.459E+04	3.166E+04
	Std	8.528E+03	6.325E+03	5.939E+03	8.612E+03	8.536E+03	3.706E+03
	RR	1	5	2	3	4	6
$f_{11}$	MV	3.943E+02	8.067E+03	2.953E+03	3.955E+03	6.356E+03	9.566E+03
	Std	1.084E+02	1.612E+04	2.645E+03	7.861E+03	9.010E+03	1.126E+04
	RR	1	5	2	3	4	6
$f_{12}$	MV	1.156E+07	5.652E+07	5.411E+07	5.501E+07	4.343E+07	5.819E+07
	Std	8.430E+06	3.285E+07	2.935E+07	3.082E+07	2.151E+07	3.343E+07
	RR	1	5	3	4	2	6
$f_{13}$	MV	9.931E+03	1.150E+04	1.428E+04	1.518E+04	8.212E+03	9.828E+03
	Std	1.141E+04	1.496E+04	1.662E+04	1.626E+04	1.091E+04	1.361E+04
	RR	3	4	5	6	1	2
$f_{14}$	MV	3.736E+05	1.212E+06	1.231E+06	1.223E+06	1.555E+06	1.047E+06
	Std	1.694E+05	6.723E+05	5.487E+05	8.810E+05	8.372E+05	5.143E+05
	RR	1	3	5	4	6	2
$f_{15}$	MV	5.062E+03	7.985E+03	7.921E+03	5.782E+03	1.388E+04	1.150E+04
	Std	5.208E+03	7.723E+03	1.152E+04	7.161E+03	1.733E+04	1.332E+04
	RR	1	4	3	2	6	5

**Table 2:** Experimental results, of the HCOA ,DLSBBPSO, TBBPSO, BBPSO, ETBBPSO and PBBPSO for $f_{16} - f_{29}$. The mean ranking points are at the end of the table.

Number	Data Type	the HCOA	DLSBBPSO	TBBPSO	BBPSO	ETBBPSO	PBBPSO
$f_{16}$	MN	5.341E+03	9.275E+03	6.487E+03	5.928E+03	6.727E+03	8.486E+03
	Std	1.670E+03	2.637E+03	2.498E+03	1.881E+03	2.549E+03	3.113E+03
	RR	1	6	3	2	4	5
$f_{17}$	MN	3.904E+03	5.815E+03	4.865E+03	4.735E+03	5.082E+03	6.341E+03
	Std	7.662E+02	1.504E+03	1.118E+03	8.836E+02	1.275E+03	1.339E+03
	RR	1	5	3	2	4	6
$f_{18}$	MN	1.410E+06	8.936E+06	4.921E+06	7.146E+06	6.481E+06	6.626E+06
	Std	7.889E+05	6.943E+06	3.290E+06	4.758E+06	4.187E+06	4.779E+06
	RR	1	6	2	5	3	4
$f_{19}$	MN	6.651E+03	1.197E+04	9.697E+03	1.010E+04	8.047E+03	8.478E+03
	Std	8.455E+03	1.317E+04	1.045E+04	1.147E+04	1.009E+04	1.244E+04
	RR	1	6	4	5	2	3
$f_{20}$	MN	3.334E+03	4.718E+03	3.790E+03	3.794E+03	3.821E+03	5.079E+03
	Std	7.455E+02	1.586E+03	1.152E+03	1.184E+03	1.373E+03	1.489E+03
	RR	1	5	2	3	4	6
$f_{21}$	MN	9.704E+02	1.067E+03	1.141E+03	1.094E+03	1.124E+03	1.115E+03
	Std	1.128E+02	1.586E+02	1.570E+02	1.693E+02	1.828E+02	1.657E+02
	RR	1	2	6	3	5	4
$f_{22}$	MN	2.382E+04	3.185E+04	2.513E+04	2.502E+04	2.492E+04	3.274E+04
	Std	8.733E+03	4.207E+03	6.240E+03	8.721E+03	7.936E+03	3.181E+03
	RR	1	5	4	3	2	6
$f_{23}$	MN	1.218E+03	1.248E+03	1.324E+03	1.253E+03	1.296E+03	1.289E+03
	Std	1.235E+02	1.029E+02	1.078E+02	1.029E+02	1.146E+02	1.399E+02
	RR	1	2	6	3	5	4
$f_{24}$	MN	1.786E+03	1.780E+03	1.884E+03	1.883E+03	1.918E+03	1.841E+03
	Std	1.527E+02	1.628E+02	1.615E+02	1.889E+02	1.792E+02	1.801E+02
	RR	2	1	5	4	6	3
$f_{25}$	MN	7.598E+02	7.528E+02	7.533E+02	7.586E+02	7.542E+02	7.609E+02
	Std	6.408E+01	6.327E+01	6.262E+01	4.453E+01	4.810E+01	6.714E+01
	RR	5	1	2	4	3	6
$f_{26}$	MN	1.335E+04	1.343E+04	1.475E+04	1.397E+04	1.458E+04	1.414E+04
	Std	1.706E+03	1.988E+03	1.800E+03	1.697E+03	1.833E+03	1.823E+03
	RR	1	2	6	3	5	4
$f_{27}$	MN	5.000E+02	5.000E+02	5.000E+02	5.000E+02	5.000E+02	5.000E+02
	Std	5.205E−04	4.631E−04	3.170E−04	4.965E−04	5.082E−04	3.539E−04
	RR	1	5	4	2	3	6
$f_{28}$	MN	5.000E+02	5.000E+02	5.000E+02	5.000E+02	5.000E+02	5.000E+02
	Std	5.282E−04	3.484E−04	3.519E−04	5.197E−04	4.428E−04	3.207E−04
	RR	1	5	4	2	3	6
$f_{29}$	MN	3.768E+03	4.203E+03	4.365E+03	4.484E+03	4.438E+03	4.442E+03
	Std	6.099E+02	8.092E+02	6.473E+02	8.220E+02	8.511E+02	9.113E+02
	RR	1	2	3	6	4	5
Average rank		1.4828	3.6207	3.9276	3.3793	3.7241	4.9655

**Table 3:** Result analysis of the HCOA, Part 1.

Function Number	Rank of the HCOA	Second best algorithm	Difference from second best algorithm	Convergence graph
2	1	BBPSO	100%	Figure 4
3	1	TBBPSO	55.81%	Figure 5
4	1	BBPSO	10.79%	Figure 6
5	1	BBPSO	16.26%	Figure 7
6	1	BBPSO	22.54%	Figure 8
8	1	DLSBBPSO	5.60%	Figure 10
10	1	TBBPSO	8.60%	Figure 12
11	1	TBBPSO	86.35%	Figure 13
12	1	BBPSO	73.39%	Figure 14
14	1	PBBPSO	64.33%	Figure 16
15	1	BBPSO	12.45%	Figure 17
16	1	BBPSO	9.90%	Figure 18
17	1	BBPSO	17.56%	Figure 19
18	1	TBBPSO	71.35%	Figure 20
19	1	ETBBPSO	17.35%	Figure 21
20	1	TBBPSO	12.01%	Figure 22
21	1	DLSBBPSO	9.04%	Figure 23
22	1	ETBBPSO	4.41%	Figure 24
23	1	DLSBBPSO	2.30%	Figure 25
26	1	DLSBBPSO	0.65%	Figure 28
27	1	BBPSO	0.00%	Figure 29
28	1	BBPSO	0.00%	Figure 30
29	1	DLSBBPSO	10.33%	Figure 31

**Table 4:** Result analysis of the HCOA, Part 1.

Number	Rank of the HCOA	Best algorithm	Difference from best algorithm	Convergence graph
1	5	DLSBBPSO	56.66%	Figure 3
7	2	DLSBBPSO	1.38%	Figure 9
9	3	DLSBBPSO	4.57%	Figure 11
13	3	ETBBPSO	4.57%	Figure 15
24	2	DLSBBPSO	0.35%	Figure 26
25	5	DLSBBPSO	0.93%	Figure 27

**Figure 3:**
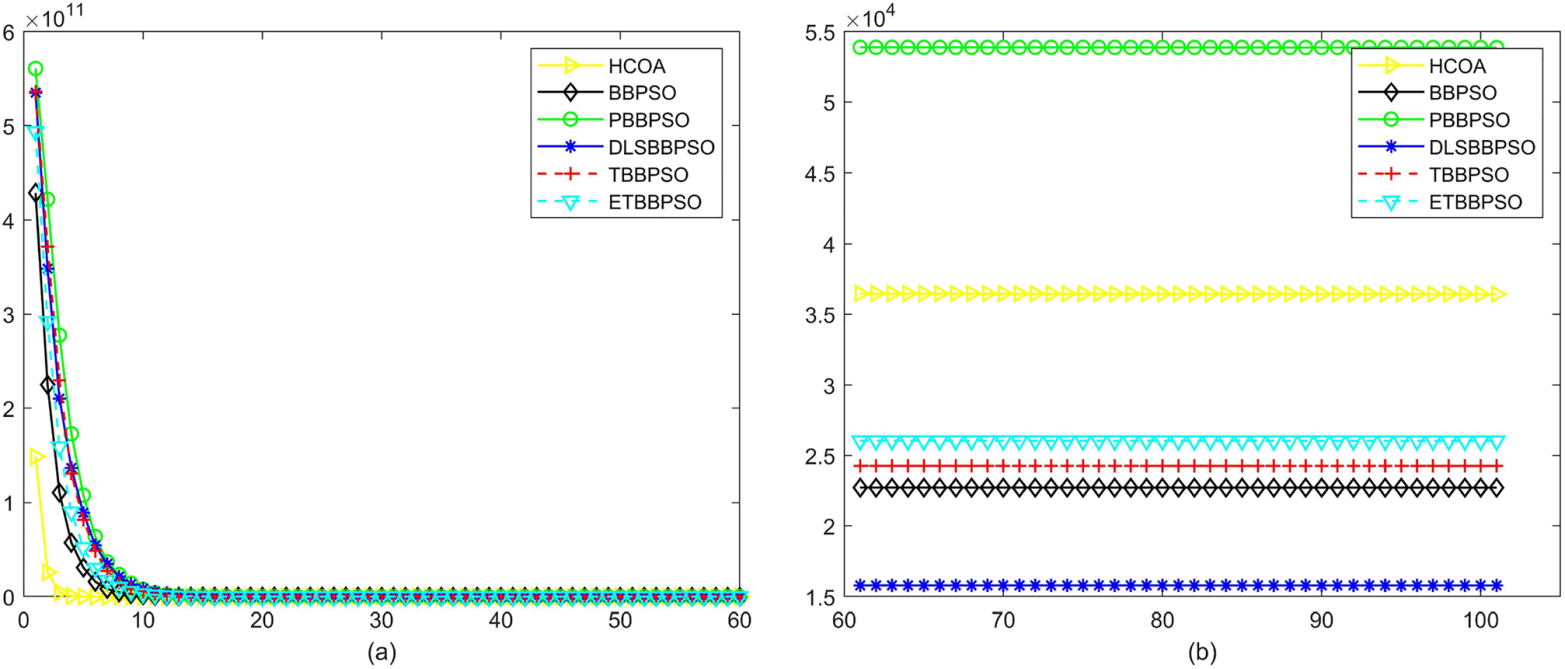
Convergence diagram, $f_{1}$, (**a**) generation 0-6*10E3, (**b**) iteration 6*10E3-10*E4.

**Figure 4:**
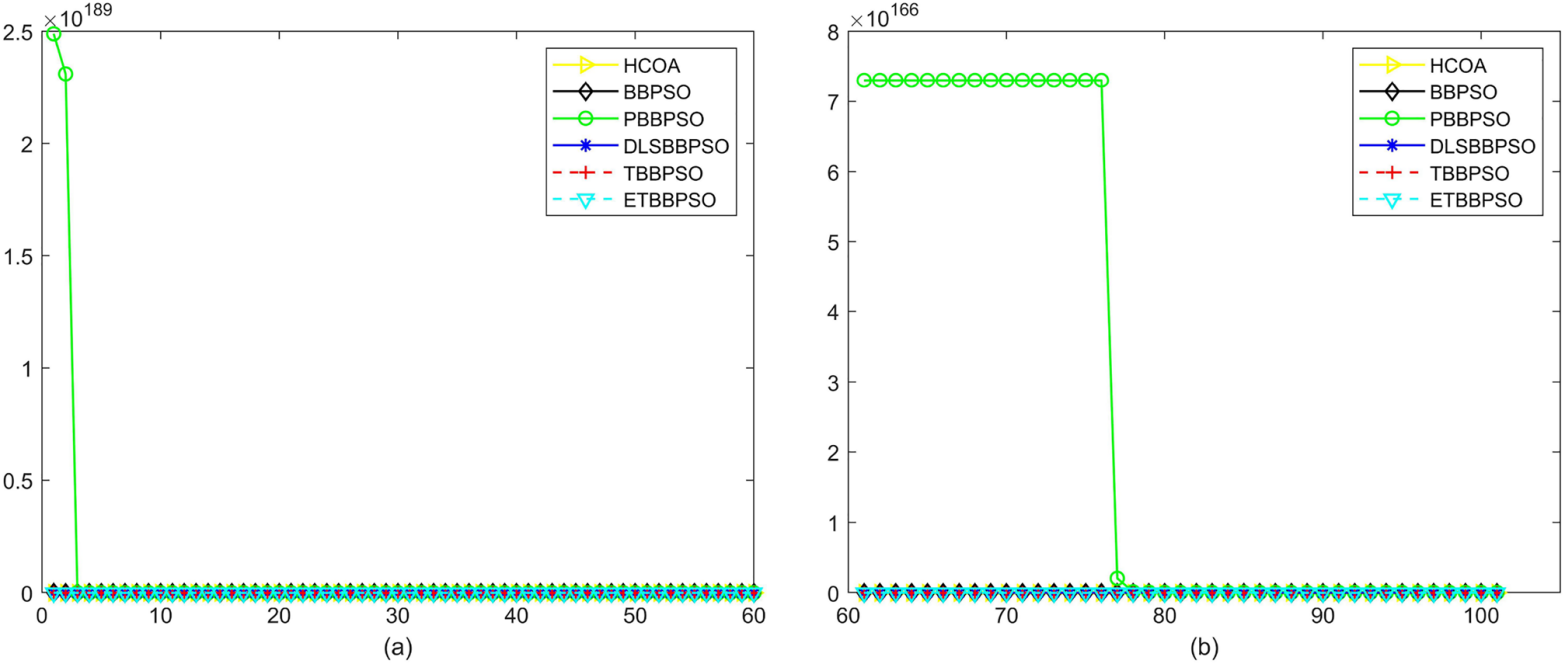
Convergence diagram, $f_{2}$,(**a**) generation 0-6*10E3, (**b**) iteration 6*10E3-10*E4.

**Figure 5:**
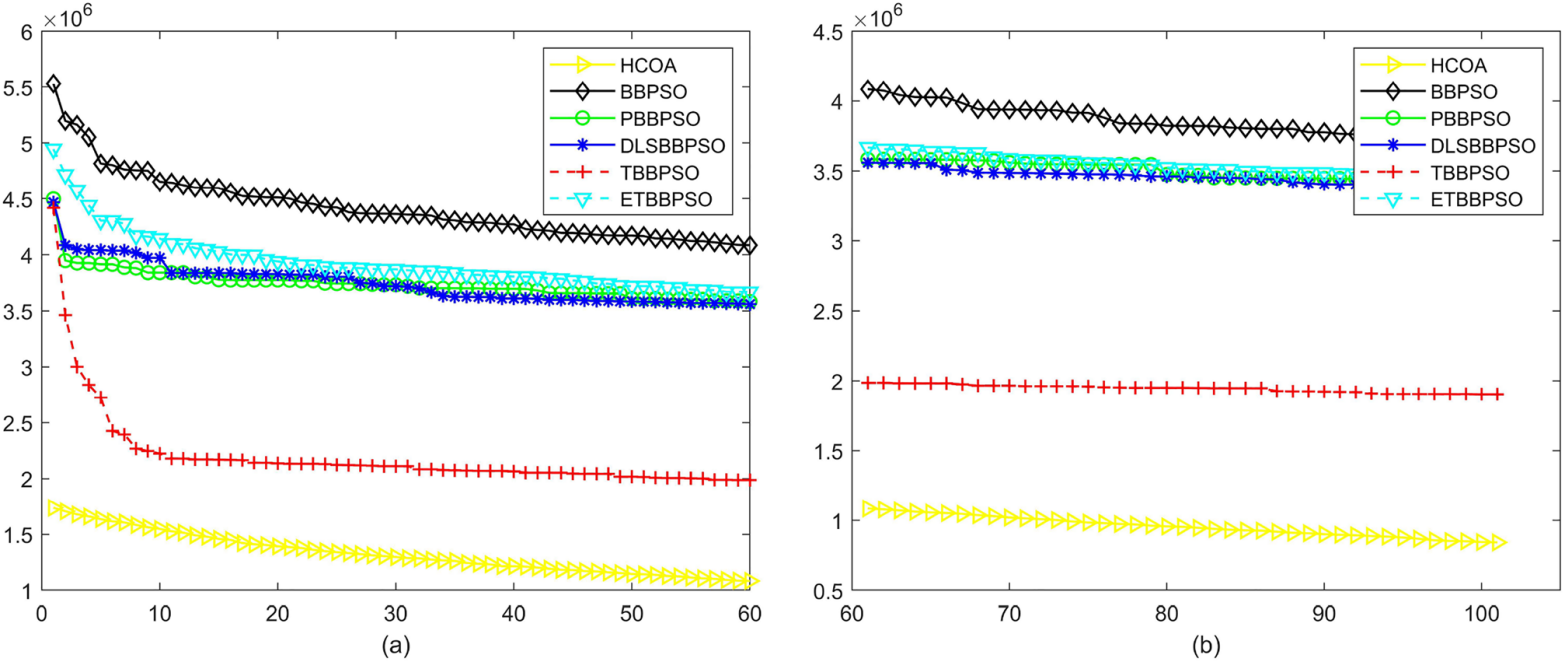
Convergence diagram, $f_{3}$,(**a**) generation 0-6*10E3, (**b**) iteration 6*10E3-10*E4.

**Figure 6:**
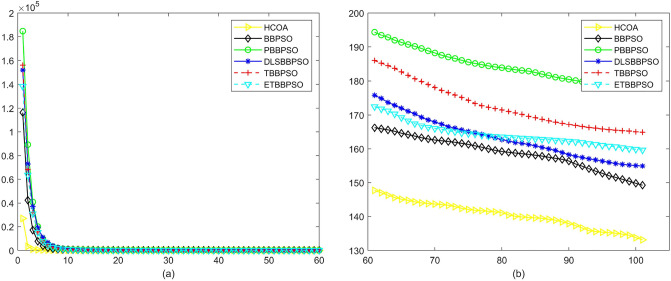
Convergence diagram, $f_{4}$,(**a**) generation 0-6*10E3, (**b**) iteration 6*10E3-10*E4.

**Figure 7:**
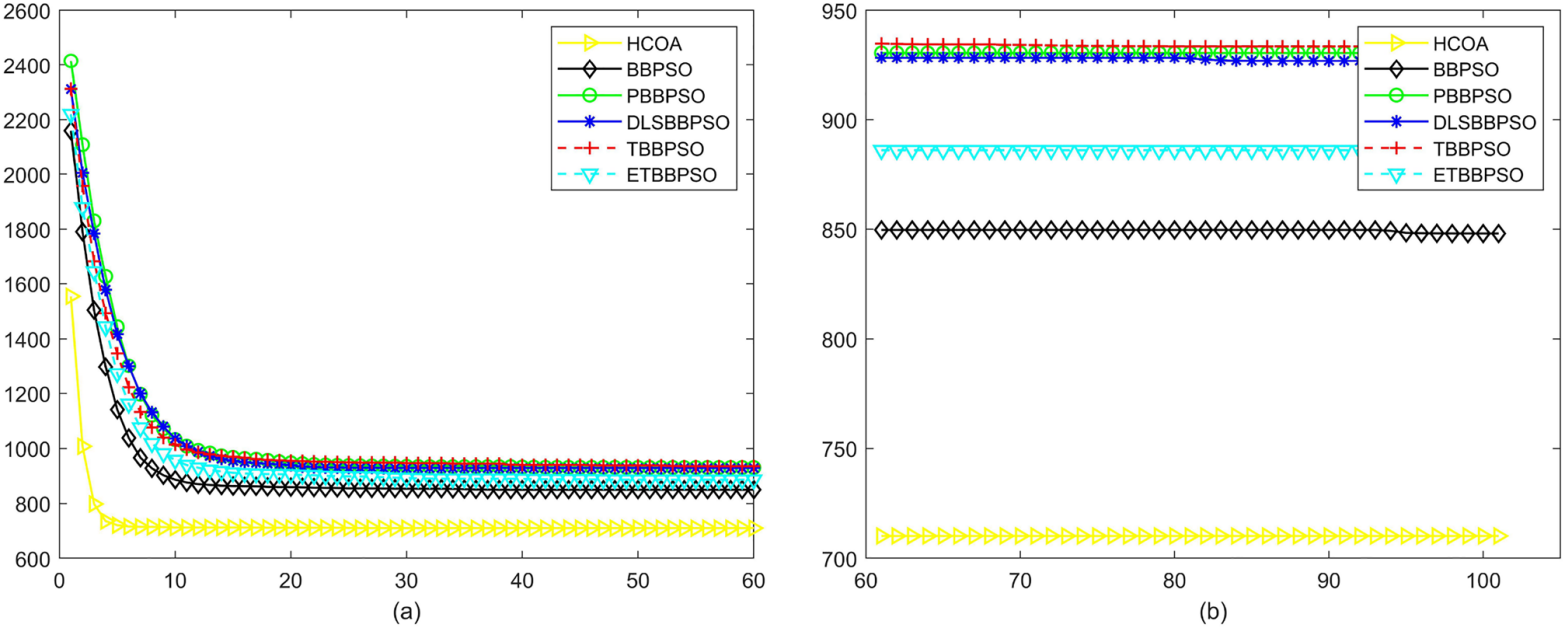
Convergence diagram, $f_{5}$,(**a**) generation 0-6*10E3, (**b**) iteration 6*10E3-10*E4.

**Figure 8:**
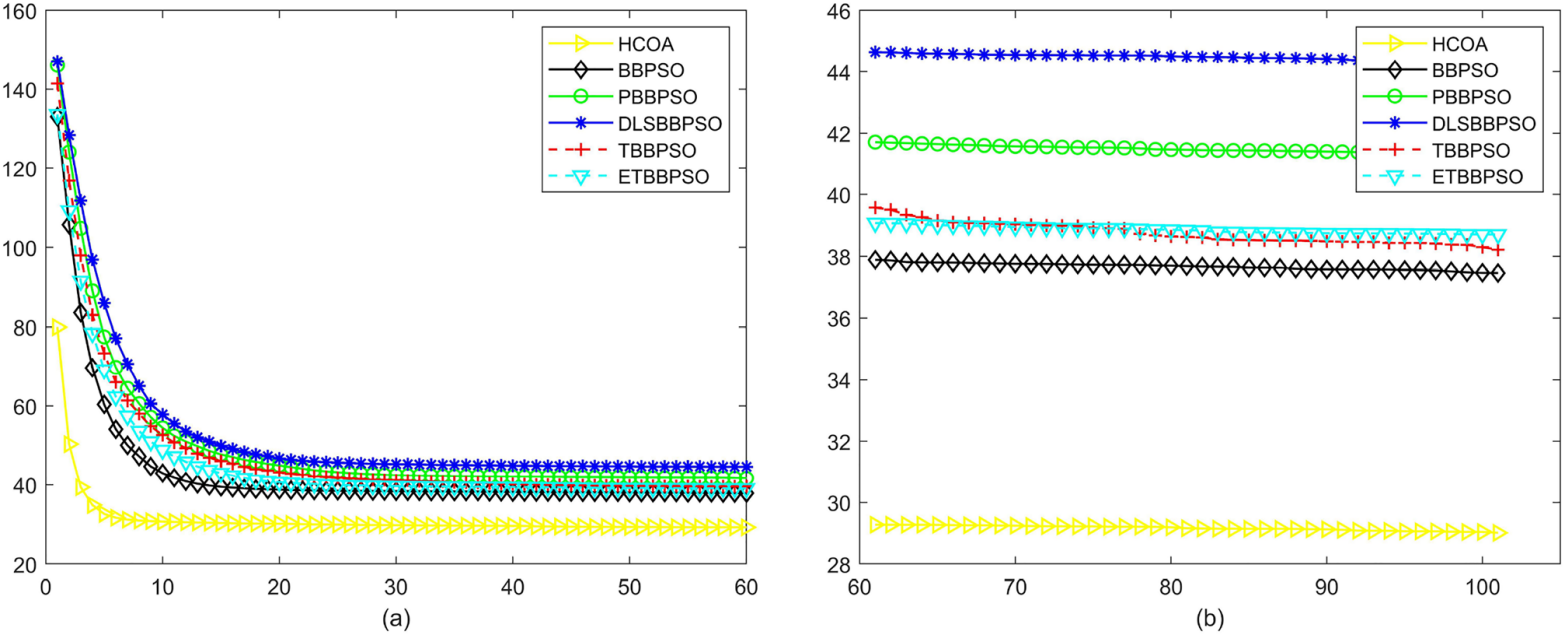
Convergence diagram, $f_{6}$,(**a**) generation 0-6*10E3, (**b**) iteration 6*10E3-10*E4.

**Figure 9:**
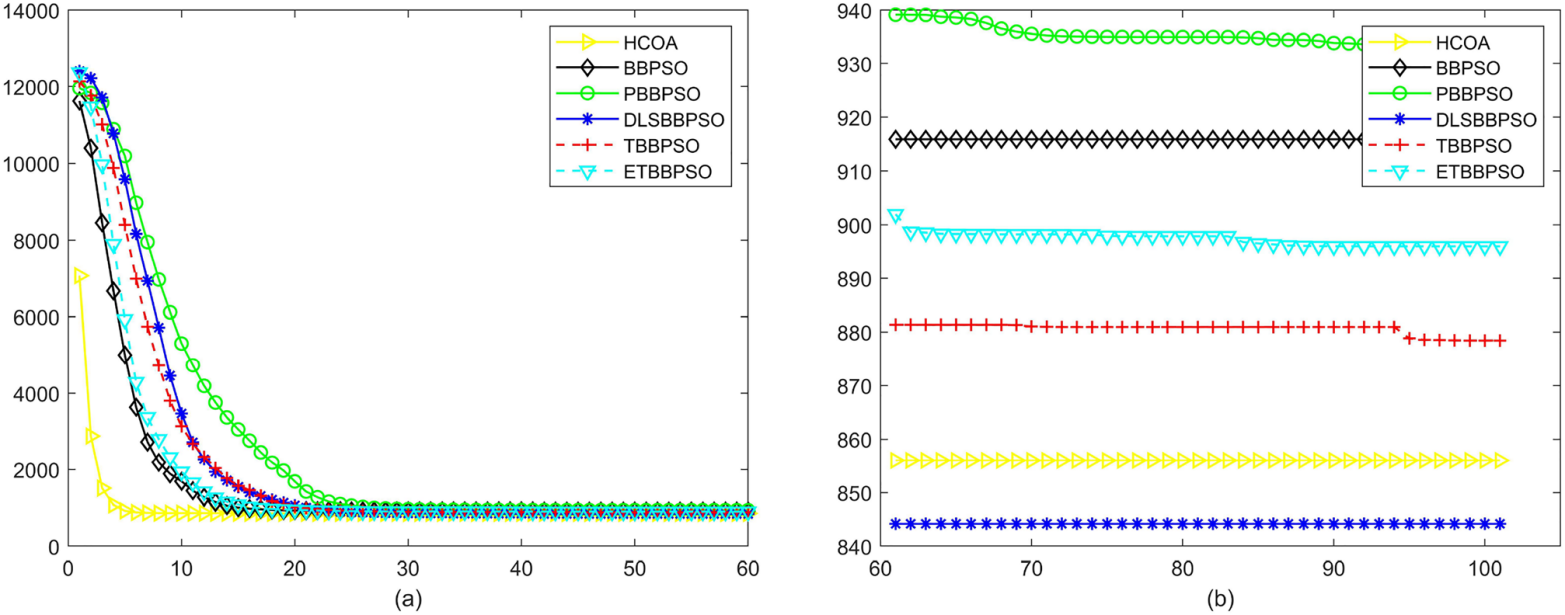
Convergence diagram, $f_{7}$,(**a**) generation 0-6*10E3, (**b**) iteration 6*10E3-10*E4.

**Figure 10:**
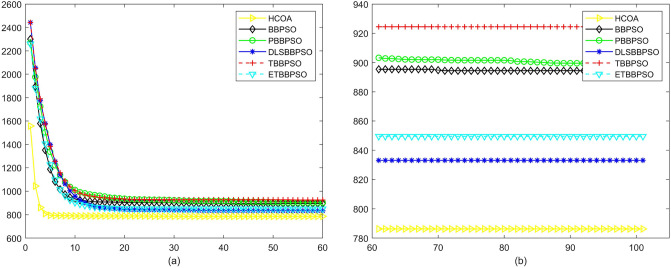
Convergence diagram, $f_{8}$,(**a**) generation 0-6*10E3, (**b**) iteration 6*10E3-10*E4.

**Figure 11:**
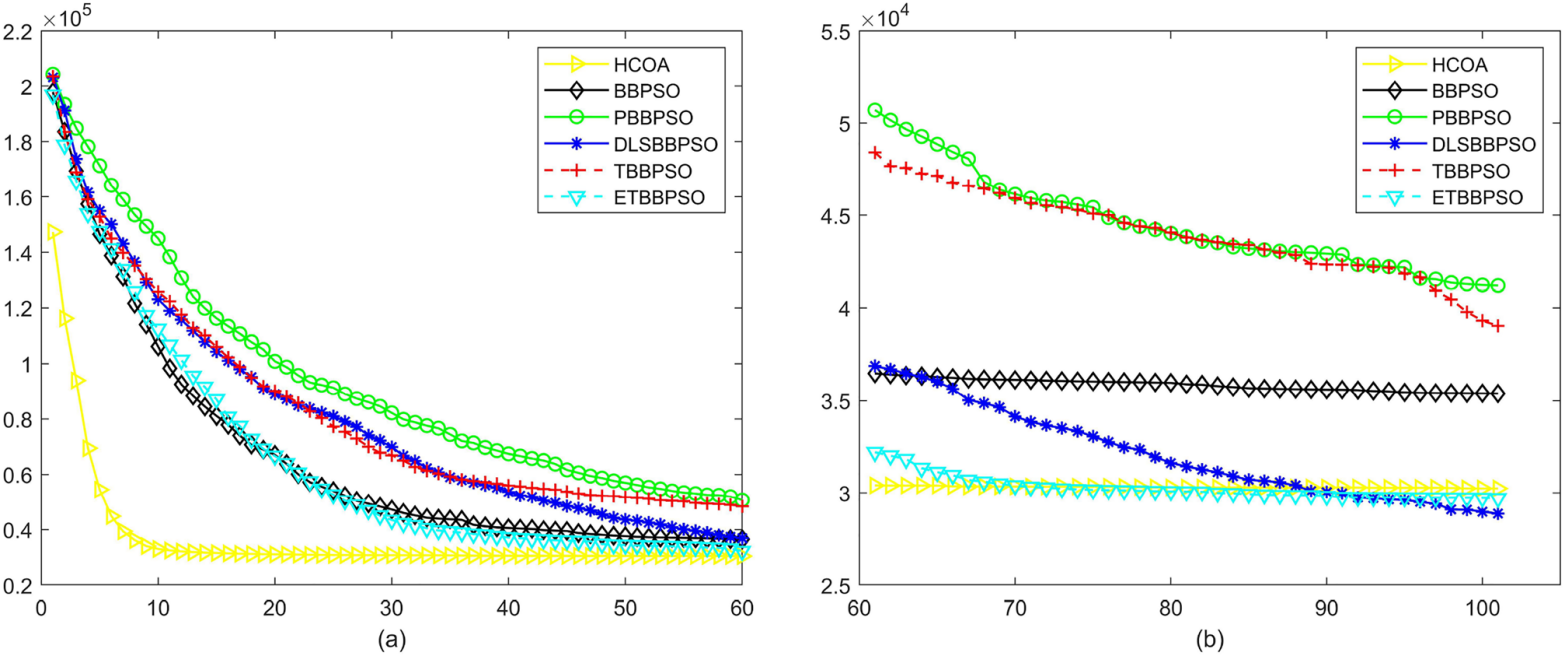
Convergence diagram, $f_{9}$,(**a**) generation 0-6*10E3, (**b**) iteration 6*10E3-10*E4.

**Figure 12:**
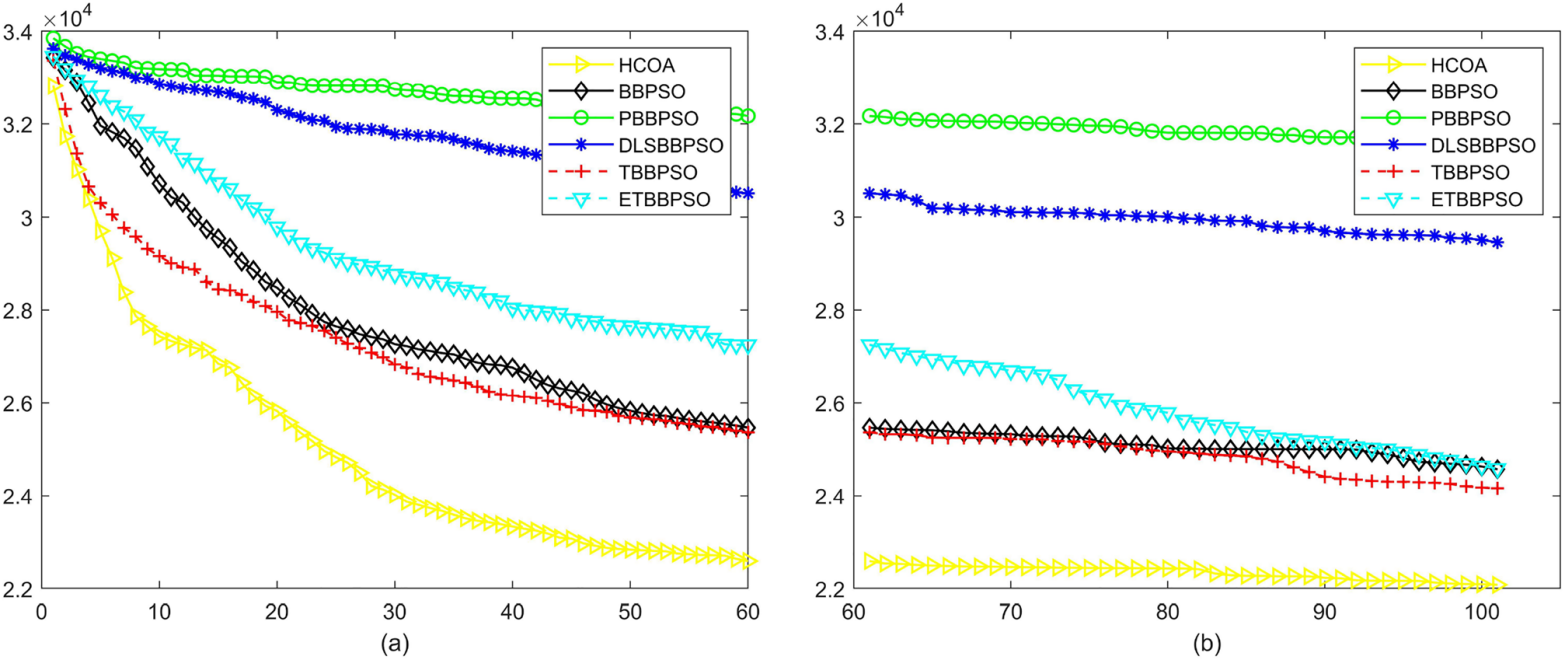
Convergence diagram, $f_{10}$,(**a**) generation 0-6*10E3, (**b**) iteration 6*10E3-10*E4.

To demonstrate the convergence performance of the HCOA, the GT in different iterations for the HCOA, BBPSO, PBBPSO, DLSBBPSO, TBBPSO and ETBBPSO are also shown in Figs. 3, 4, 5, 6, 7, 8, 9, 10, 11, 12, 13, 14, 15, 16, 17, 18, 19, 20, 21, 22, 23, 24, 25, 26, 27, 28, 29, 30 and 31. The scale on the vertical axis represents the value of CE. The horizontal coordinate denotes the number of generations, and the vertical coordinate denotes the value of GT.

**Figure 13:**
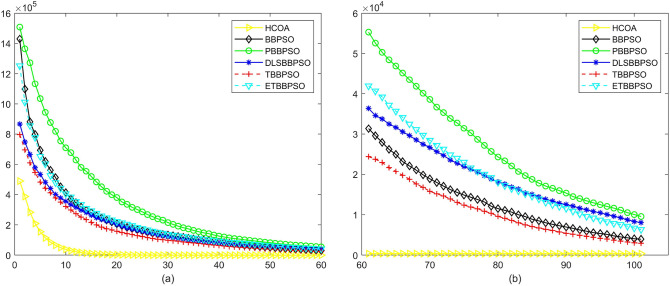
Convergence diagram, $f_{11}$,(**a**) generation 0-6*10E3, (**b**) iteration 6*10E3-10*E4.

**Figure 14:**
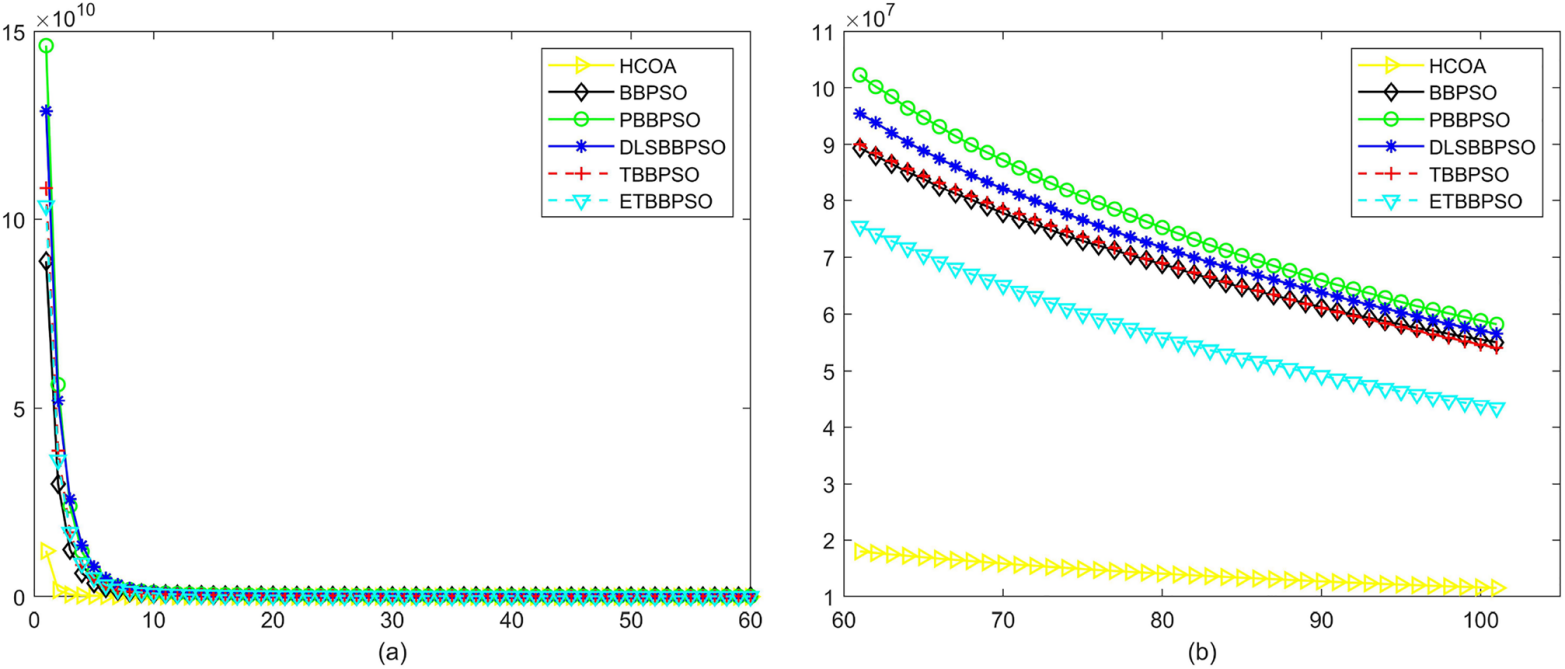
Convergence diagram, $f_{12}$,(**a**) generation 0-6*10E3, (**b**) iteration 6*10E3-10*E4.

**Figure 15:**
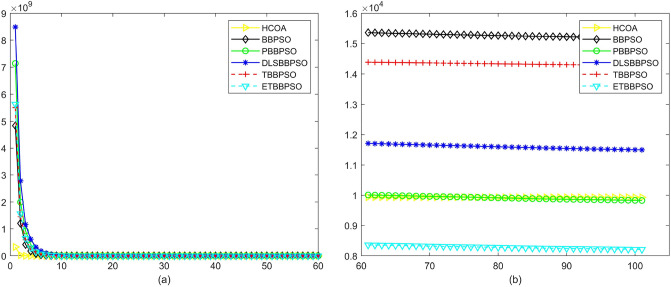
Convergence diagram, $f_{13}$,(**a**) generation 0-6*10E3, (**b**) iteration 6*10E3-10*E4.

**Figure 16:**
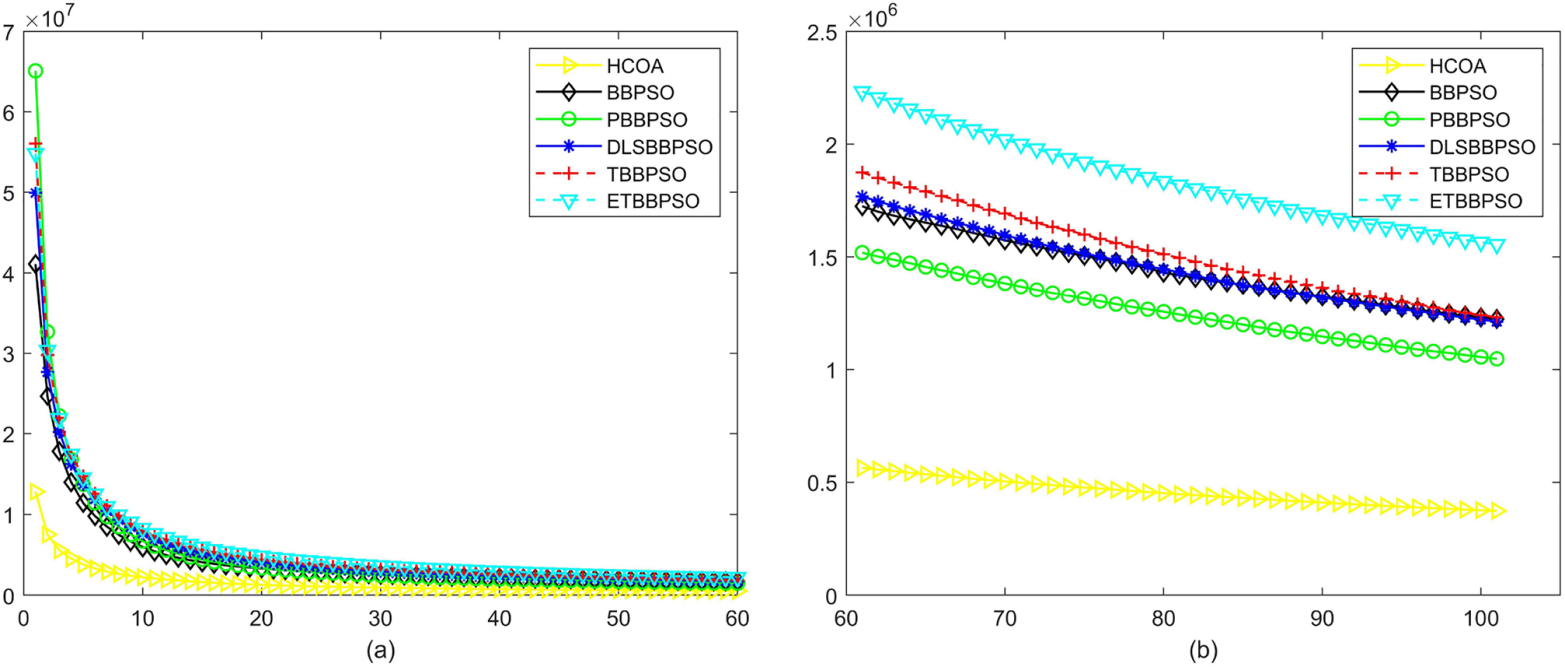
Convergence diagram, $f_{14}$,(**a**) generation 0-6*10E3, (**b**) iteration 6*10E3-10*E4.

**Figure 17:**
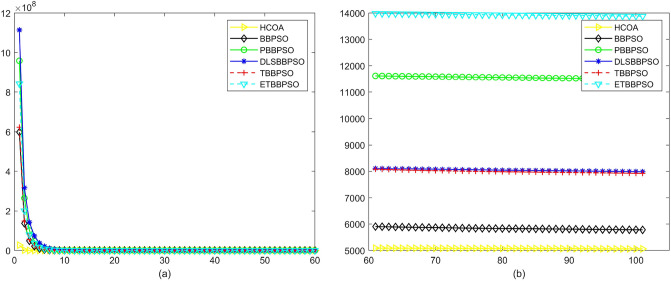
Convergence diagram, $f_{15}$,(**a**) generation 0-6*10E3, (**b**) iteration 6*10E3-10*E4.

**Figure 18:**
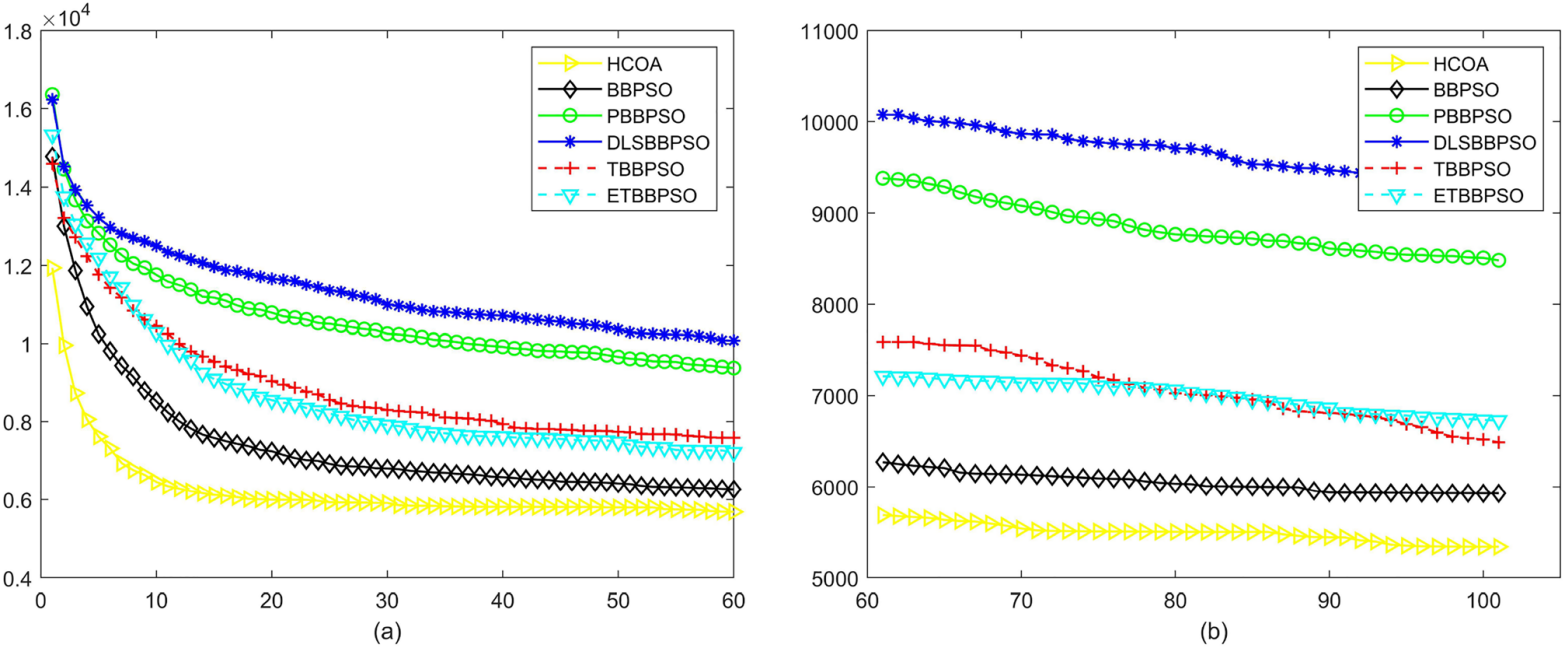
Convergence diagram, $f_{16}$,(**a**) generation 0-6*10E3, (**b**) iteration 6*10E3-10*E4.

**Figure 19:**
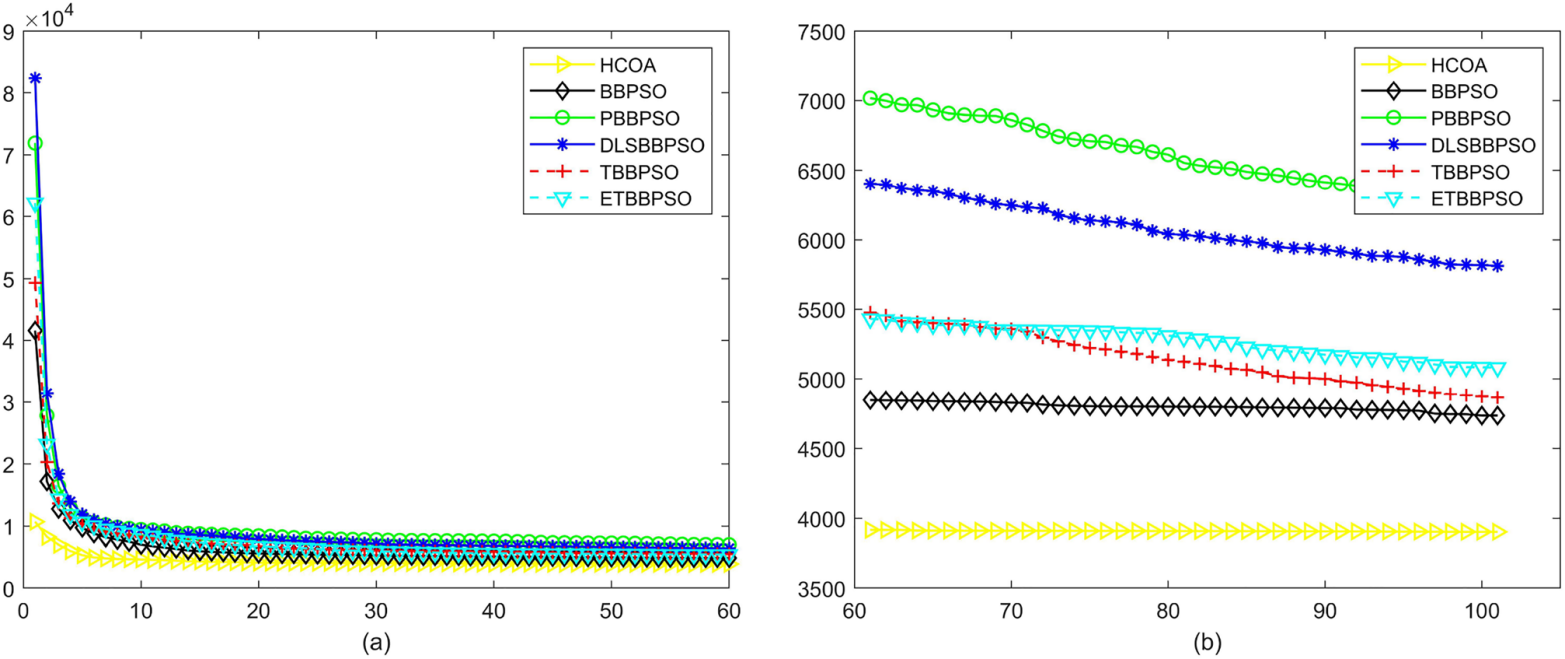
Convergence diagram, $f_{17}$,(**a**) generation 0-6*10E3, (**b**) iteration 6*10E3-10*E4.

**Figure 20:**
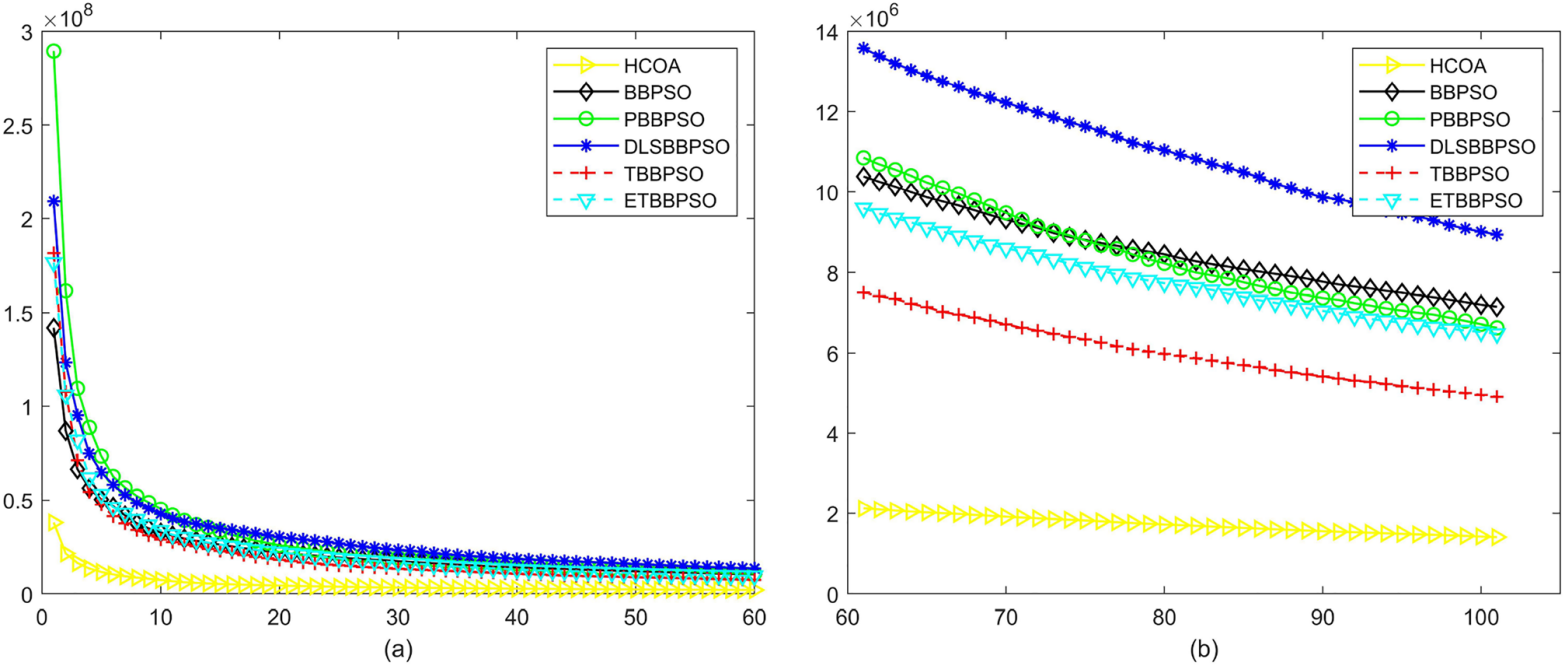
Convergence diagram, $f_{18}$,(**a**) generation 0-6*10E3, (**b**) iteration 6*10E3-10*E4.

After comparing and counting, HCAO ranks first in the number of functions among the 29 benchmarked functions in CEC2017 with 23, ranked second, third, and fifth with two each, and none ranked fourth and sixth. And the ranking shows that HCAO has a better performance at simple multipeak function (f3–f9), hybrid function (f10–f19), and composition function than other algorithms.

According to Figs. 3, 4, 5, 6, 7, 8, 9, 10, 11, 12, 13, 14, 15, 16, 17, 18, 19, 20, 21, 22, 23, 24, 25, 26, 27, 28, 29, 30 and 31, except for *f*1, *f*8, *f*9, *f*13, *f*19, *f*24 and *f*25, the HCOA is significantly better than the other algorithms in terms of convergence speed and accuracy. The time complexity of the five optimization algorithms for the HCOA and the control group are linearly transformed by addition and subtraction without changing the order of magnitude of the time complexity. Therefore, the time complexity of the HCOA and the other optimization algorithms are the same *O*(*n*).

**Figure 21:**
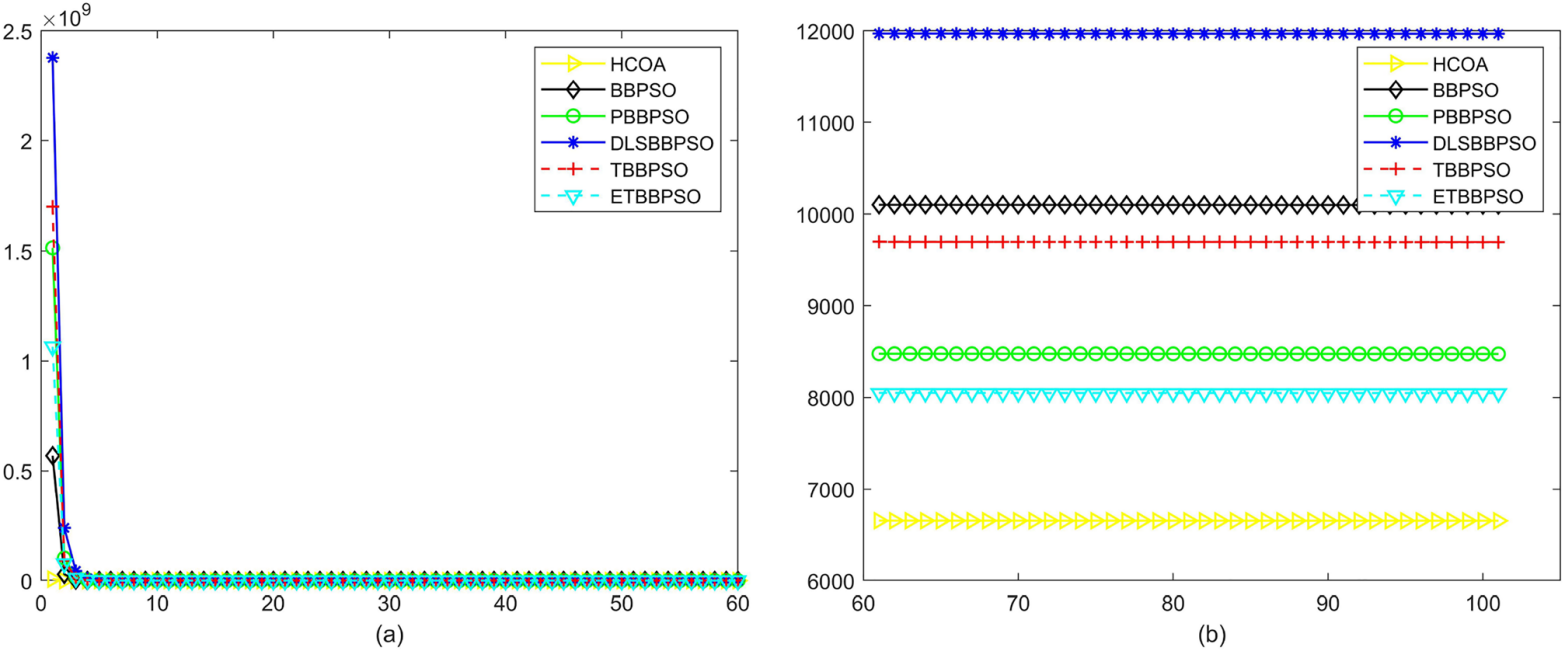
Convergence diagram, $f_{19}$,(**a**) generation 0-6*10E3, (**b**) iteration 6*10E3-10*E4.

**Figure 22:**
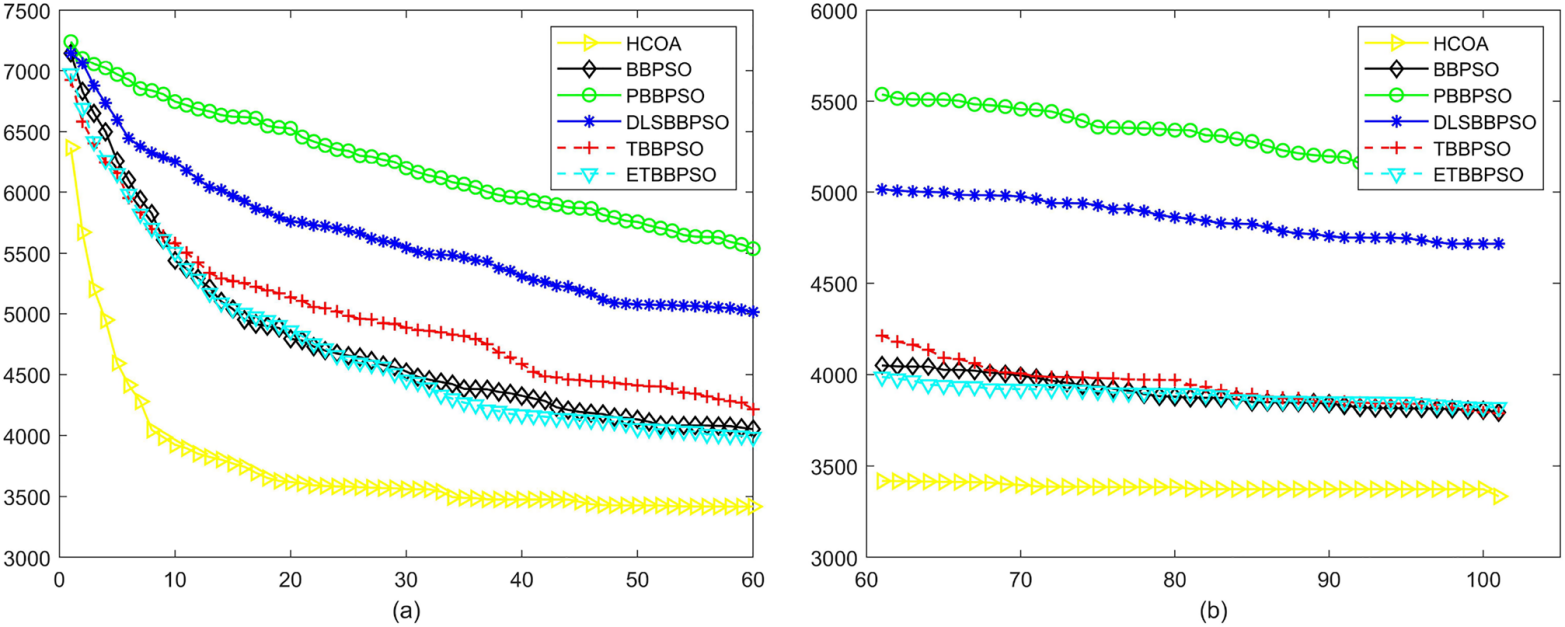
Convergence diagram, $f_{20}$,(**a**) generation 0-6*10E3, (**b**) iteration 6*10E3-10*E4.

**Figure 23:**
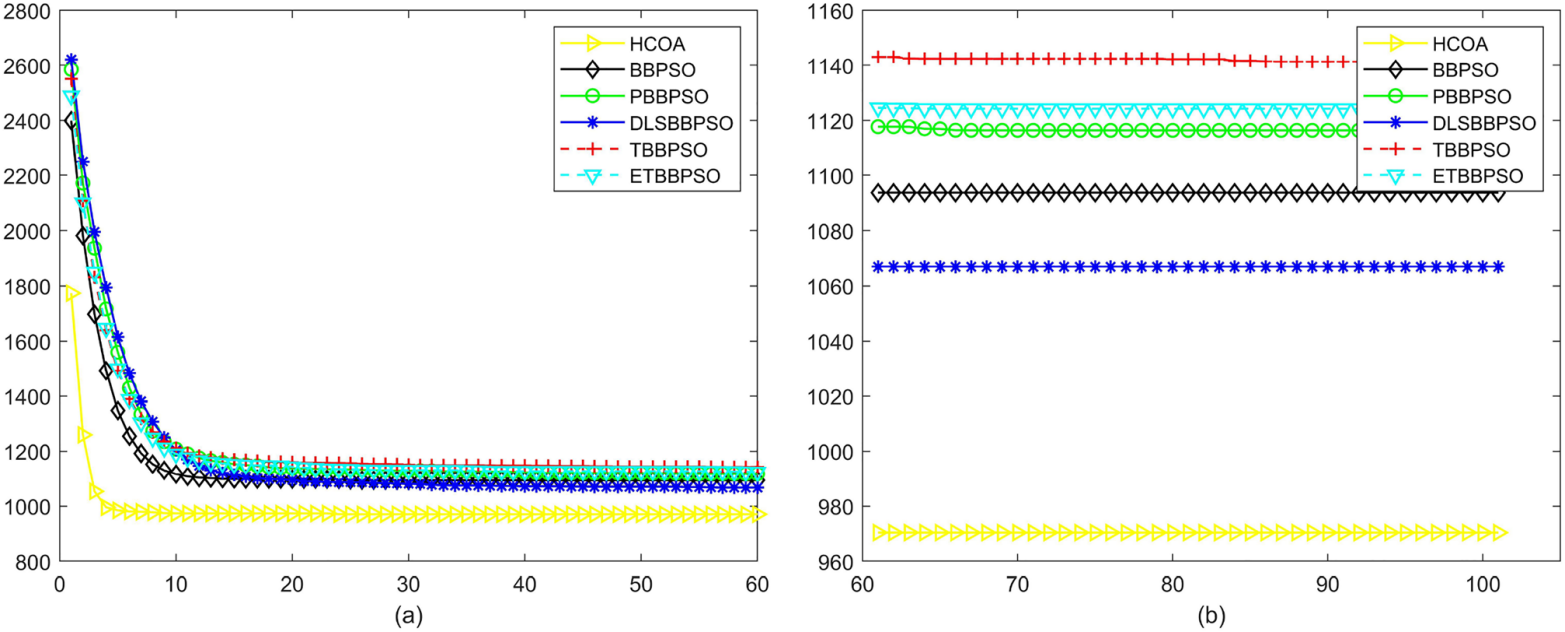
Convergence diagram, $f_{21}$,(**a**) generation 0-6*10E3, (**b**) iteration 6*10E3-10*E4.

**Figure 24:**
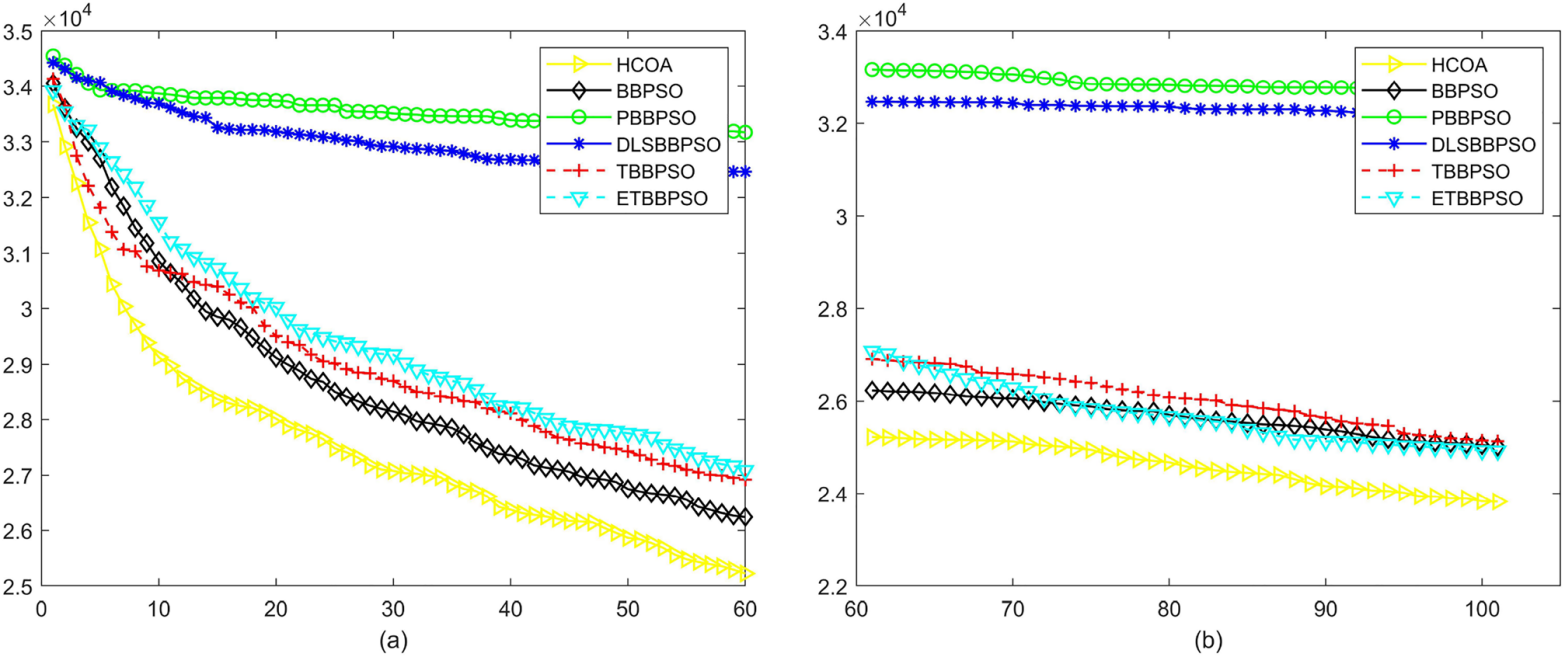
Convergence diagram, $f_{22}$,(**a**) generation 0-6*10E3, (**b**) iteration 6*10E3-10*E4.

**Figure 25:**
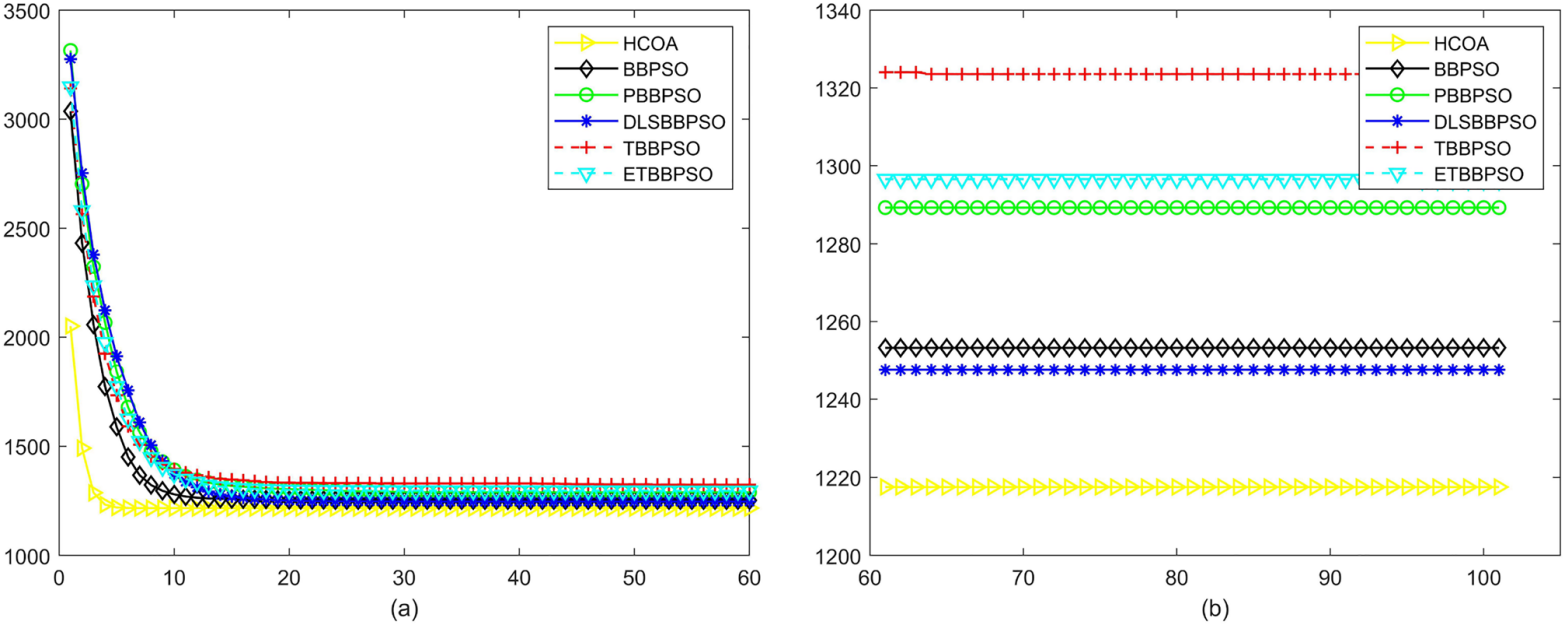
Convergence diagram, $f_{23}$,(**a**) generation 0-6*10E3, (**b**) iteration 6*10E3-10*E4.

**Figure 26:**
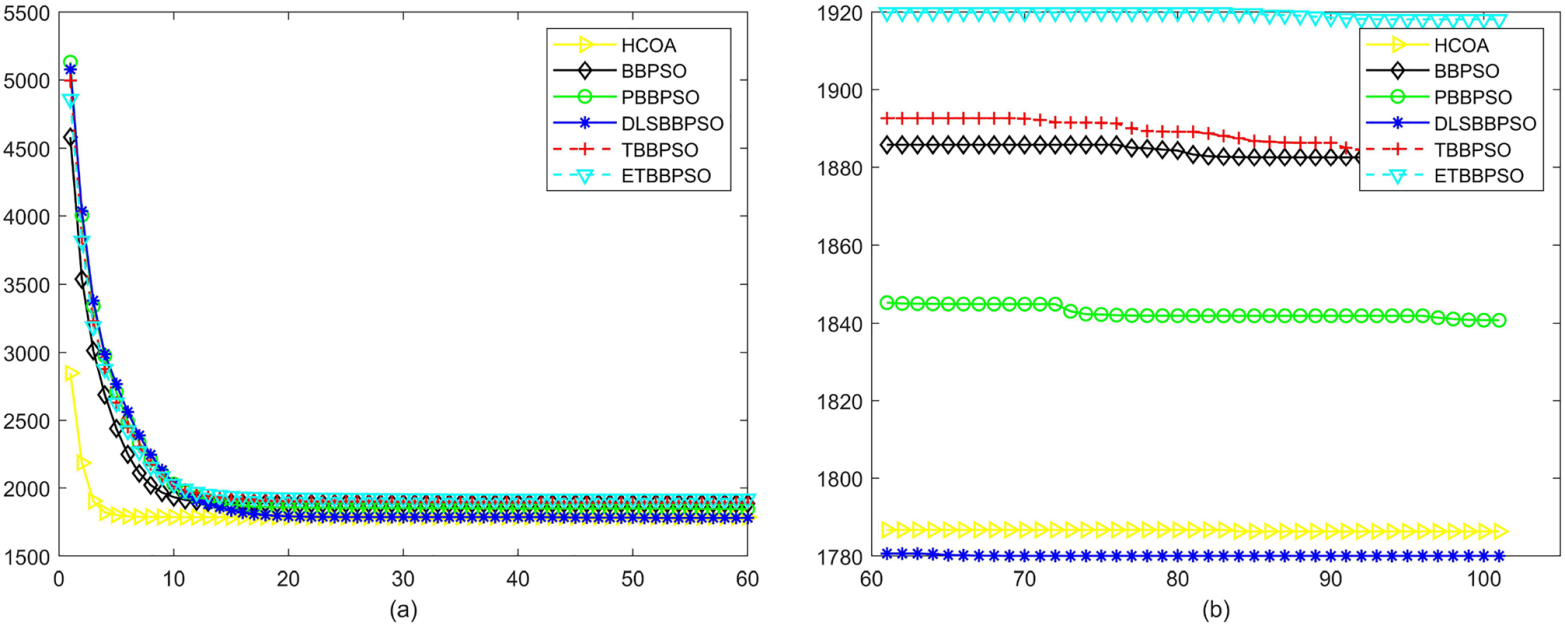
Convergence diagram, $f_{24}$,(**a**) generation 0-6*10E3, (**b**) iteration 6*10E3-10*E4.

**Figure 27:**
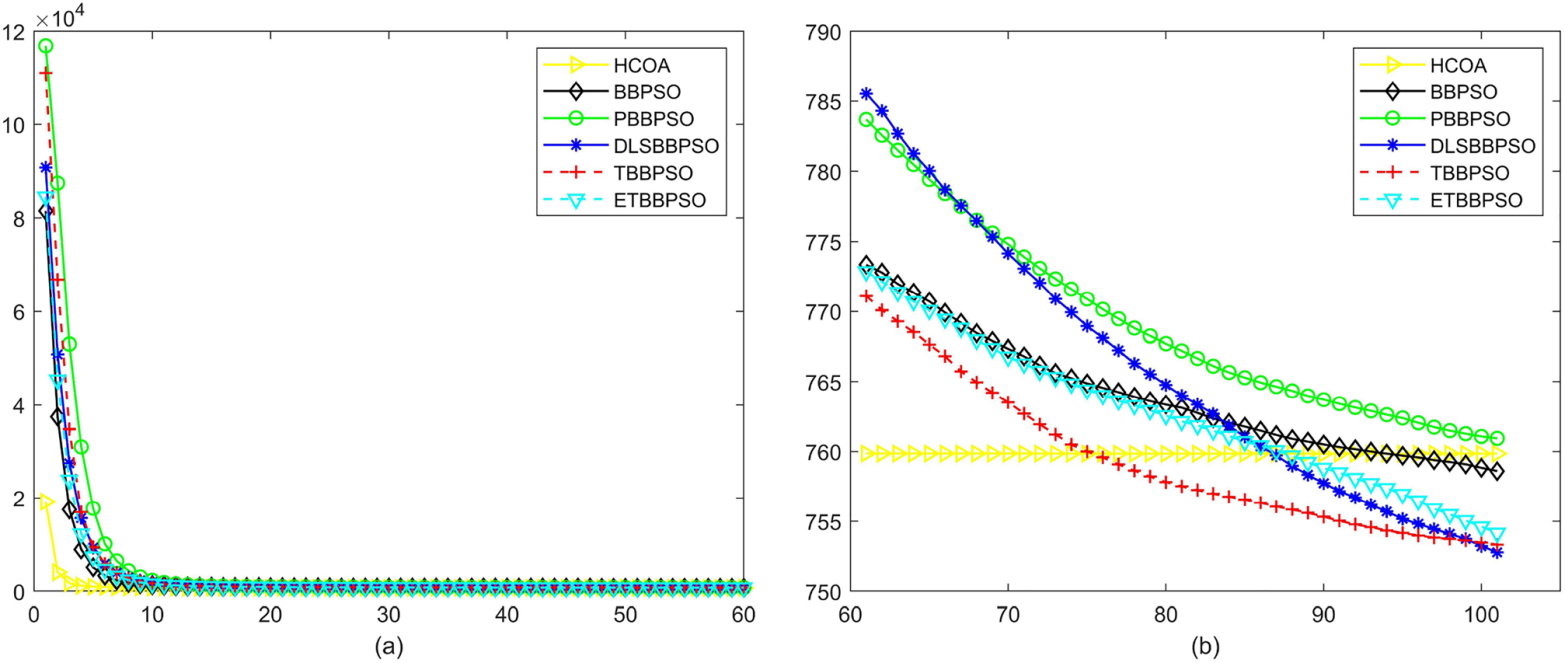
Convergence diagram, $f_{25}$,(**a**) generation 0-6*10E3, (**b**) iteration 6*10E3-10*E4.

### Comparison with the new parameter-free algorithm

To further prove the superiority of the HCOA algorithm in high-dimensional optimization problems, we choose the state-of-the-art method, the BPSO-CM^40^ algorithm, as the control group, to conduct experiments on the highest dimension of 100 recommended by CEC2017. To minimize the effect of chance errors on the experimental results, all the trials are attempted 37 times with a population size of 100 and a maximum number of iterations of 1.00E+4. In addition, the overall effectiveness (OE) of the HCOA and BPSO-CM is computed by the results in Tables 5 and 6. The OE is calculated by Eq. (4).

4 $$\begin{aligned} OE=\frac{N-L}{N}*100\% \end{aligned}$$

where *N* is the number of benchmark functions, and *L* represents the target algorithm loss in the competition. The OE results are shown in Table 5. The results indicate that the HCOA has the best performance.

**Figure 28:**
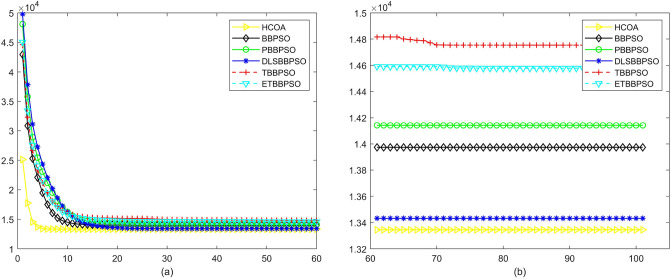
Convergence diagram, $f_{26}$,(**a**) generation 0-6*10E3, (**b**) iteration 6*10E3-10*E4.

**Figure 29:**
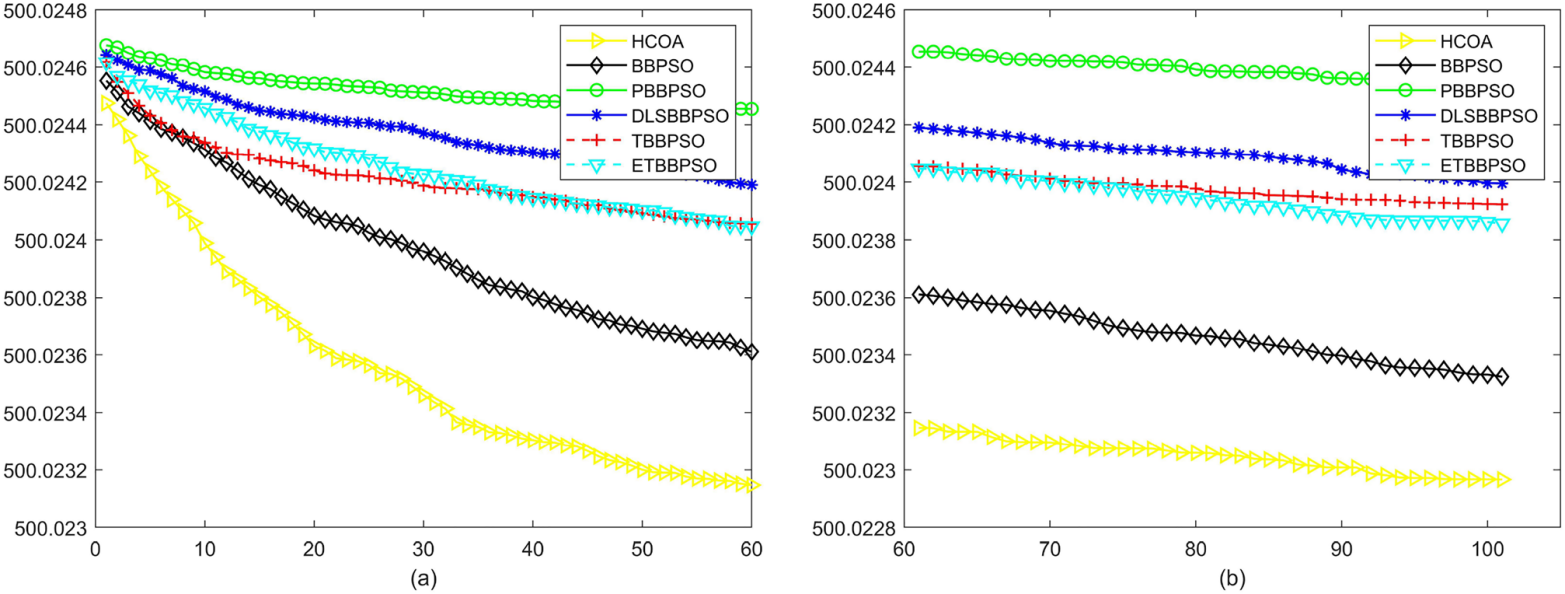
Convergence diagram, $f_{27}$,(**a**) generation 0-6*10E3, (**b**) iteration 6*10E3-10*E4.

**Figure 30:**
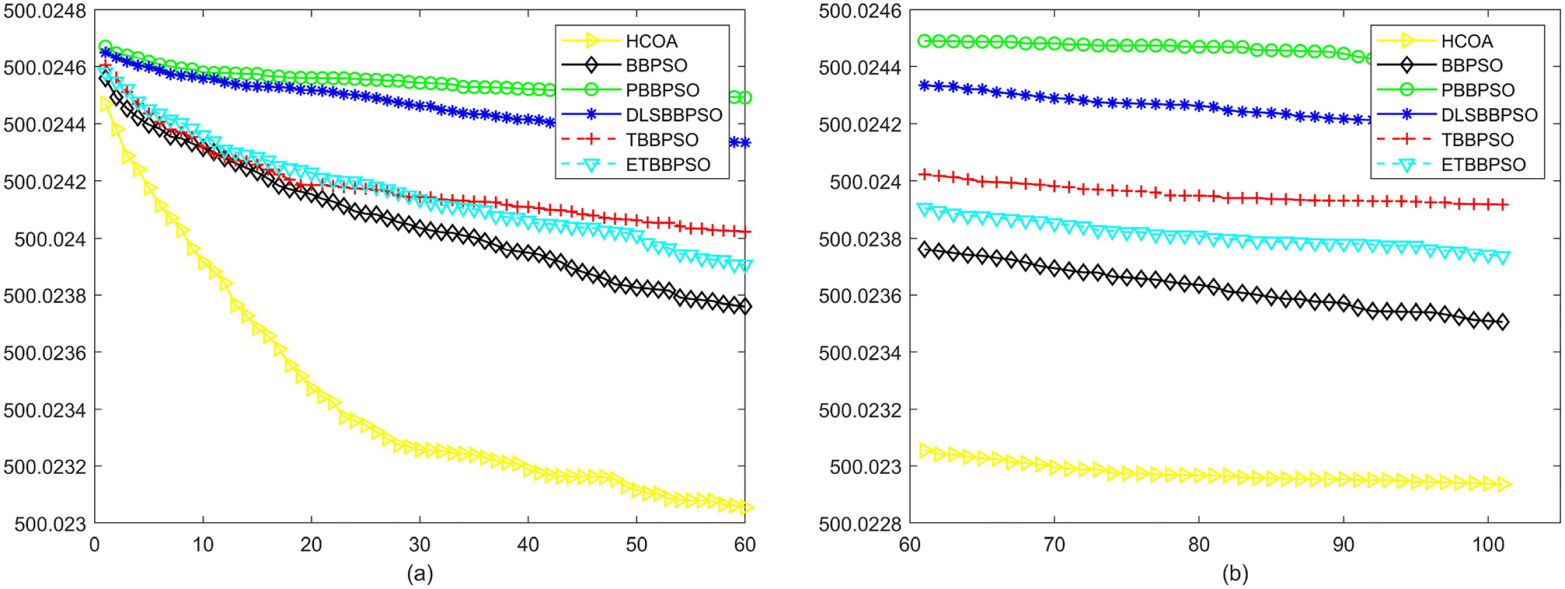
Convergence diagram, $f_{28}$,(**a**) generation 0-6*10E3, (**b**) iteration 6*10E3-10*E4.

**Figure 31:**
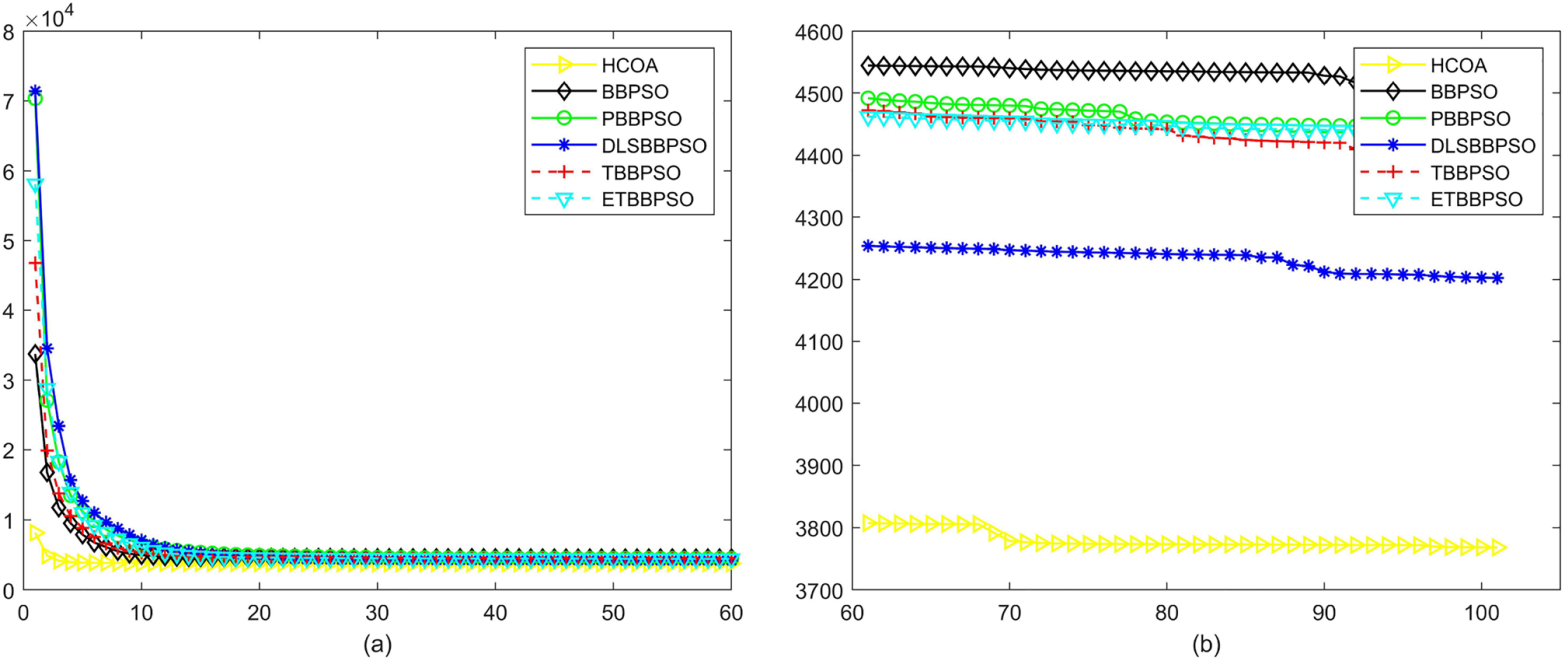
Convergence diagram, $f_{29}$,(**a**) generation 0-6*10E3, (**b**) iteration 6*10E3-10*E4.

**Table 5:** Experimental Results of the HCOA and BPSO-CM for $f_1 - f_{15}$.

Function	The HCOA		BPSO-CM	
1	MV	Std	MV	Std
	3.645E+04	5.445E+04	2.799E+04	2.839E+04
2	MV	Std	MV	Std
	2.707E+90	1.647E+91	1.456E+97	8.859E+97
3	MV	Std	MV	Std
	8.421E+05	4.333E+05	1.453E+06	2.252E+06
4	MV	Std	MV	Std
	1.331E+02	5.133E+01	1.383E+02	4.179E+01
5	MV	Std	MV	Std
	7.102E+02	1.438E+02	7.889E+02	1.452E+02
6	MV	Std	MV	Std
	2.901E+01	7.219E+00	3.514E+01	9.023E+00
7	MV	Std	MV	Std
	8.560E+02	1.552E+02	8.802E+02	1.332E+02
8	MV	Std	MV	Std
	7.863E+02	1.464E+02	7.629E+02	1.351E+02
9	MV	Std	MV	Std
	3.024E+04	7.992E+03	3.342E+04	9.051E+03
10	MV	Std	MV	Std
	2.208E+04	8.528E+03	1.679E+04	6.507E+03
11	MV	Std	MV	Std
	3.943E+02	1.084E+02	4.258E+02	1.349E+02
12	MV	Std	MV	Std
	1.156E+07	8.430E+06	1.550E+07	1.136E+07
13	MV	Std	MV	Std
	9.931E+03	1.141E+04	9.101E+03	9.633E+03
14	MV	Std	MV	Std
	3.736E+05	1.694E+05	3.910E+05	2.398E+05
15	MV	Std	MV	Std
	5.062E+03	5.208E+03	9.967E+03	1.726E+04

**Table 6:** Experimental Results of the HCOA and BPSO-CM for $f_16 - f_{29}$. The OE are at the end of the table.

Function	The HCOA		BPSO-MC	
16	MV	Std	MV	Std
	5.341E+03	1.670E+03	5.223E+03	1.189E+03
17	MV	Std	MV	Std
	3.904E+03	7.662E+02	4.450E+03	8.408E+02
18	MV	Std	MV	Std
	1.410E+06	7.889E+05	1.385E+06	8.217E+05
19	MV	Std	MV	Std
	6.651E+03	8.455E+03	7.050E+03	6.119E+03
20	MV	Std	MV	Std
	3.334E+03	7.455E+02	3.226E+03	5.726E+02
21	MV	Std	MV	Std
	9.704E+02	1.128E+02	1.008E+03	1.382E+02
22	MV	Std	MV	Std
	2.382E+04	8.733E+03	1.952E+04	7.287E+03
23	MV	Std	MV	Std
	1.218E+03	1.235E+02	1.237E+03	1.232E+02
24	MV	Std	MV	Std
	1.786E+03	1.527E+02	1.768E+03	1.913E+02
25	MV	Std	MV	Std
	7.598E+02	6.408E+01	7.723E+02	5.521E+01
26	MV	Std	MV	Std
	1.335E+04	1.706E+03	1.336E+04	1.886E+03
27	MV	Std	MV	Std
	5.000E+02	5.205E−04	5.000E+02	3.802E−04
28	MV	Std	MV	Std
	5.000E+02	5.282E−04	5.000E+02	3.109E−04
29	MV	Std	MV	Std
	3.768E+03	6.099E+02	3.799E+03	7.298E+02
OE	68.97%		31.03%	

It can be seen from Tables 5 and 6 that the HCOA performs better than BPSO-CM in 20 functions. Meanwhile, the OE of the HCOA reaches 68.97%, which is 37.94% higher than the 31.03% of BPSO-CM. The experimental results show that the HCOA can provide a high precision solution for single objective high-dimensional optimization problems.

## Conclusions

A novel hermit crab optimization algorithm (the HCOA) that produces high-precision results for high-dimensional optimization problems is proposed in this paper. the HCOA achieves high-accuracy resolution of single-objective optimization problems by modelling the behaviour of hermit crab populations. The optimal search and the historical path search are used in the HCOA to balance the depth search and breadth search. The cooperation of the optimal search and historical path search achieves high-precision optimization in high-dimensional spaces. Moreover, both the optimal search and the historical path search have linear computation times, which means that the time complexity of the HCOA is *O*(*n*).

In the experimental part of this paper, the CEC2017 benchmark functions are used. In a total of 29 test functions, the HCOA scores 23 firsts. Compared with the state-of-the-art BBPSO-based method, BPSO-CM and the HCOA win 20 of 29 tests. All the experimental results demonstrate that the HCOA generates highly accurate and robust results for high-dimensional optimization problems.

However, the HCOA cannot be applied to multiobjective optimization problems and single-objective noncontinuous optimization problems. Furthermore, in the unimodal functions of CEC2017, the performance of the HCOA is inferior to that of BPSO-CM. Therefore, one of the main future research directions is applying the HCOA to multiobjective optimization problems. Additionally, due to the linear time complexity, combining the HCOA with other famous evolutionary strategies, such as SE and PSO, to achieve higher accuracy and greater robustness is another solid option.

## Data Availability

All data generated or analysed during this study are included in this published article and https://github.com/GuoJia-Lab-AI/crab.
